# Ready References to Amendments, Revocations, Modifications, Explanations, Etc.

**Published:** 1918-12

**Authors:** 


### READY REFERENCES TO AMENDMENTS, REVOCATIONS, MODIFICATIONS, EXPLANATIONS, ETC.

1917 — 1918

Corrected to :

G. O. 167, October 1st, 1918.

Bul. 74, Sept. 27th, 1918.

Prepared by

REFERENCE LIBRARY,

Finance and Accounting Division.

C. S. O. A. E. F.

### G. H. Q. GENERAL ORDERS

### PERTAINING TO MEDICAL SERVICE IN THE AMERICAN EXPEDITIONARY FORCES

| Year. | G. O. No. | Section. | Paragraph. | REMARKS | G. O. No. | Section. | Paragraph. | Date. |
|---|---|---|---|---|---|---|---|---|
| 1917 | 2 | | 2 | Modified by | 24 | 3 | | 8/21/17 |
| " | 2 | 1 | | Amended by | 67 | 1 | 9 | 11/30/17 |
| " | 2 | 2 | | Amplified by | 31 | | | 2/16/18 |
| " | 2 | 3 | | Amplified by | 79 | 1 | 4-6 | 12/30/17 |
| " | 2 | | | Partly amended by | 100 | | 7 | 6/20/18 |
| " | 3 | | | Revoked by | 13 | | 1 | 7/13/17 |
| " | 5 | | | Revoked by | 13 | | 1 | 7/13/17 |
| " | 6 | | | Supplementing re Venereal Disease by | 34 | 1 | | 9/9/17 |
| " | 6 | | | Supplementing re Venereal Disease by | 77 | | | 12/18/17 |
| " | 7 | | | Attention directed re destruction of beehives by | 42 | 3 | | 3/14/18 |
| " | 8 | | | Revoked by | 31 | | | 2/16/18 |
| " | 13 | | | Revoked by | 146 | | | 9/1/18 |
| " | 16 | | | Supplemented by | 10 | 3 | | 1/16/18 |
| " | 18 | | 3 | Revoked by | 41 | 4 | | 9/24/17 |
| " | 18 | 2 | | Revoked by | 50 | 2 | | 3/30/18 |
| " | 19 | | 1 | Amended by | 67 | 1 | 9 | 11/30/17 |
| " | 19 | | 1 | Partly Amended by | 100 | | 7 | 6/20/18 |
| " | 19 | 1 | | Supplemented by | 40 | 1 | 2 | 3/13/18 |
| " | 19 | 4 | | Supplemented by W.D.-G.O. No. 7 and by | 37 | 3 | | 3/7/18 |
| " | 20 | 3 | 4 | Amended by | 66 | 3 | | 11/27/17 |
| " | 20 | | | Amended by | 75 | | | 12/14/17 |
| " | 20 | | | Amplified by | 31 | | | 2/16/18 |
| " | 21 | | | If conflicting, revoked by | 30 | 5 | | 2/15/18 |
| " | 21 | | | Amended by | 30 | 4 | 7-m | 2/15/18 |
| " | 21 | 6 | | If conflicting, revoked by | 91 | 2 | | 6/10/18 |
| " | 22 | | 1 | Revoked by | 15 | 1 | | 1/24/18 |
| " | 22 | 3 | | Modified by | 154 | 3 | | 9/12/18 |
| " | 23 | | | Supplemented by | 7 | 1 | 6 | 1/9/18 |
| " | 23 | 4 | 1 | Supplemented by | 75 | | | 12/24/17 |
| " | 23 | 4 | 2 | Supplemented by | 46 | | | 3/26/18 |
| " | 23 | 1 | 5 | Supplemented by | 105 | 1 | 1 | 6/28/18 |
| " | 24 | | | Amplified by | 31 | | | 2/16/18 |

| Year. | G. O. No. | Section. | Paragraph. | REMARKS | G. O. No. | Section. | Paragraph. | Date. |
|---|---|---|---|---|---|---|---|---|
| 1917 | 25 | 2 | | Amplified by | 31 | | | 2/16/18 |
| ” | 27 | | | Revoked by | 30 | 5 | | 2/15/18 |
| ” | 29 | | | If conflicting, revoked by | 111 | 11 | 15 | |
| ” | 30 | | | If conflicting, revoked by | 111 | 11 | 15 | 7/8/18 7/8/18 |
| ” | 35 | 1 | | Supplemented by | 45 | 2 | 1 | 3/25/18 |
| ” | 36 | | 3 | Re Correspondants' Uniform Revoked by | 96 | 1 | 4 | 6/15/18 |
| ” | 37 | | | Amplified by | 31 | | | 2/16/18 |
| ” | 38 | | 9 | Amended by | 19 | 2 | | 1/31/18 |
| ” | 38 | | 11-12 | Amended by | 37 | 1 | 1-2 | 3/7/18 |
| ” | 38 | 1 | 2 | Supplemented by | 31 | 1 | 3 | 1/21/18 |
| ” | 38 | 1 | 10 | Supplemented by | 41 | 7 | | 3/14/18 |
| ” | 38 | 2 | | Rescinded by | 105 | 3 | 1 | 6/28/18 |
| ” | 39 | | | Revoked by | 122 | 2 | 1 | 7/26/18 |
| ” | 40 | 2 | 3-4 | Explained by | 57 | 2 | | 11/9/17 |
| ” | 40 | 2 | 7 | Amended by | 2 | 3 | | 1/13/18 |
| ” | 40 | 1 | 1 | Supplemented by | 19 | 1 | 1-2 | 1/31/18 |
| ” | 40 | 1 | 2 | Amplified by | 22 | | | 2/5/18 |
| ” | 40 | 1 | 2-a | Supplemented by | 8 | | | 1/10/18 |
| ” | 41 | 5 | 4 | Explained by | 46 | 4 | 1 | 10/10/17 |
| ” | 41 | 4 | 5 | Revoked by | 80 | 2 | 1 | 12/21/17 |
| ” | 41 | 2 | 2 | Amplified by | 31 | | | 2/16/18 |
| ” | 42 | 2 | 2 | Amended by | 63 | 4 | | 4/27/18 |
| ” | 43 | | | Revoked by | 44 | | | 3/23/18 |
| ” | 44 | 2 | | Modified by | 154 | 3 | | 9/12/18 |
| ” | 44 | 5 | 4 | Amended by | 71 | 2 | 1 | 12/10/17 |
| ” | 44 | 5 | 3 | Supplemented by | 46 | | | 3/26/18 |
| ” | 44 | 5 | 4 | Supplemented by | 41 | | | 3/14/18 |
| ” | 45 | | | Supplemented by | 35 | | | 3/5/18 |
| ” | 46 | 1 | 3-c | Amended by | 156 | 2 | | 9/14/18 |
| ” | 46 | 3 | 6 | Revoked by | 36 | 1 | | 3/6/18 |
| ” | 46 | 2 | | Revoked by | 80 | 1 | 4 | 12/21/17 |
| ” | 46 | 3 | 1 | Amended by | 54 | 2 | | 4/8/18 |
| ” | 46 | 1 | Sub. 3 | Amended by | 58 | 1 | 1 | 4/18/18 |
| ” | 48 | 1 | | Supplemented by | 15 | 2 | | 1/24/18 |
| ” | 50 | | | If conflicting, revoked by | 5 | 1 | 8 | 1/7/18 |
| ” | 51 | | | Change in name of Art. School by | 93 | 2 | | 6/11/18 |
| ” | 53 | | 2 | Amended by | 78 | 2 | | 5/25/18 |
| ” | 54 | 3 | | Supplemented by | 68 | 1 | 1-2 | 12/3/17 |
| ” | 54 | 3 | | Supplemented by | 15 | 2 | | 1/24/18 |
| ” | 56 | | | Supplemented by | 5 | | | 1/7/18 |
| ” | 58 | | | Amended, designation of Medical Officer by | 88 | 1 | 2 | 6/6/18 |
| ” | 58 | | | Supplemented by | 102 | | 6 | 6/25/18 |
| ” | 60 | | 3 | Supplemented by | 159 | 4 | | 9/19/18 |
| ” | 62 | | | Supplemented by | 24 | 2 | | 2/8/18 |
| ” | 62 | | | Supplemented by | 45 | 1 | | 3/25/18 |
| ” | 63 | | 4 | Explained by | 33 | 5 | | 2/20/18 |

| Year. | G. O. No. | Section. | Paragraph. | REMARKS | G. O. No. | Section. | Paragraph. | Date. |
|---|---|---|---|---|---|---|---|---|
| 1917 | 63 | | 3 | Identity cards at embarkation, revoked by | 42 | 1 | | 3/14/18 |
| " | 63 | 3 | | Supplemented by | 25 | 1 | 1 | 2/9/18 |
| " | 64 | 3 | | Re Corps, revoked by | 9 | 1 | 5 | 1/15/18 |
| " | 65 | 4 | | Amended by | 78 | 2 | | 12/18/17 |
| " | 66 | 3 | | Amended by | 75 | | | 12/14/17 |
| " | 66 | 4 | | If conflicting, revoked by | 32 | 3 | 1-d | 2/18/18 |
| " | 67 | | | Revoked by | 100 | | 15 | 6/20/18 |
| " | 70 | | | Revoked by | 74 | | | 5/11/18 |
| " | 71 | | 5 | Revoked by | 43 | 2 | 1 | 3/22/18 |
| " | 71 | 2 | 1 | Supplemented by | 41 | 1 | | 3/14/18 |
| " | 71 | | | If conflicting, revoked by | 111 | 11 | 15 | 7/8/18 |
| " | 74 | 1 | 1 | Enlarged by | 167 | 4 | 1 | 10/1/18 |
| " | 74 | 2 | | Supplemented by | 41 | 2 | | 3/14/18 |
| " | 73 | | | Revoked by | 44 | | | 3/23/18 |
| " | 79 | 1 | 1 | Amplified by | 31 | | | 2/16/18 |
| " | 79 | 1 | 6 | Supplemented by | 40 | | | 3/13/18 |
| " | 80 | 2 | 2 | Amended by | 50 | 3 | | 3/20/18 |
| " | 80 | 2 | 2 | Modified by | 157 | 1 | | 9/15/18 |
| " | 81 | | | Amended, designation of Medical Officer by | 88 | 1 | 2 | 6/6/18 |
| " | 82 | | | Partly revoked by | 91 | 1 | 5 | 6/10/18 |
| 1918 | 3 | 2 | | Amended by | 38 | 3 | | 3/9/18 |
| " | 6 | | | Re Arms, Suspended by | 15 | 3 | | 1/24/18 |
| " | 6 | | 21 | Extended by | 29 | 3 | | 2/14/18 |
| " | 6 | | | If conflicting, revoked by | 38 | 1 | 4 | 3/9/18 |
| " | 6 | | 9 | Amended by | 135 | 2 | | 8/17/18 |
| " | 7 | | 8 | Revoked by | 148 | 1 | | 9/4/18 |
| " | 10 | 3 | 7-8-9 10-10a | Amended by | 73 | 2 | 1 | 5/10/18 |
| " | 10 | | 3 Sub-7 | Revoked by | 28 | 2 | | 2/13/18 |
| " | 11 | 3 | 4 | Amended by | 50 | 4 | 2-E(5) | 3/30/18 |
| " | 12 | | 1 | Revoked by | 49 | | | 3/29/18 |
| " | 14 | 4 | | Modified by | 34 | 3 | 2 | 2/25/18 |
| " | 15 | 1 | | Amended by | 78 | 3 | | 5/25/18 |
| " | 15 | | | Amended by | 121 | 7 | | 7/25/18 |
| " | 15 | 1 | | If conflicting, revoked by | 146 | | | 9/1/18 |
| " | 18 | 4 | | Revoked by | 58 | 6 | | 4/18/18 |
| " | 18 | | 35-56-61-62-66 | Amended by | 60 | 1 | | 4/20/18 |
| " | 18 | | 51-52 | Re Billets, amended by | 50 | 4 | 2-E(4) | 3/30/18 |
| " | 18 | | | Amended by | 48 | 3 | | 3/28/18 |
| " | 19 | 2 | | Amended by | 50 | 2 | | 3/30/18 |
| " | 21 | 3 | | Supplemented by | 130 | 2 | 2 | 8/6/18 |
| " | 22 | | | Revoked by | 117 | 1 | 1 | 7/16/18 |
| " | 23 | 3 | | Revoked by | 68 | 1 | 1 | 5/4/18 |
| " | 24 | | | Revoked by | 144 | 1 | 2 | 8/29/18 |

| Year. | G. O. No. | Section. | Paragraph. | REMARKS | G. O. No. | Section. | Paragraph. | Date. |
|---|---|---|---|---|---|---|---|---|
| 1918 | 29 | 2 | 1-B | Extended by | 50 | 4 | 2-A | 3/30/18 |
| " | 30 | 4 | N-2 | Amended by | 53 | 2 | | 4/7/18 |
| " | 30 | 2 | | Re triplicate reports of burial, revoked by | 89 | 2 | | 6/7/18 |
| " | 30 | 4 | 7-N | If conflicting, revoked by | 91 | 2 | | 6/10/18 |
| " | 30 | 4 | 7-F | Re making reports of burial, amended by | 100 | | 14 | 6/20/18 |
| " | 30 | 4 | 7-0 | Explained by | 101 | | | 6/24/18 |
| " | 31 | | Table 4 | Re Pyrotechnics, amended by | 48 | 2 | | 3/28/18 |
| " | 31 | | | Extended by | 68 | 2 | 1 | 5/4/18 |
| " | 31 | | | Explained by | 64 | 1 | 1 | 4/29/18 |
| " | 31 | | | Amended by | 64 | 2 | | 4/29/18 |
| " | 31 | | | If conflicting, revoked by | 111 | 11 | 15 | 7/8/18 |
| " | 31 | | | Re Service of Utilities, revoked by | 114 | 1 | 1 | 7/11/18 |
| " | 31 | | | Re Chief Tank Corps, amended by | 121 | 1 | 1 | 7/25/18 |
| " | 31 | | | Supplemented by | 131 | 2 | E | 8/7/18 |
| " | 31 | | | Re Remount Service, amended by | 139 | 2 | 1 | 8/24/18 |
| " | 31 | | | Re Chief Engineer, supplemented by | 144 | 4 | 1 | 8/29/18 |
| " | 32 | | | Re Artillery Candidates, amended by | 45 | 3 | 2 | 3/25/18 |
| " | 32 | 3 | 2 | Amended by | 131 | 5 | | 8/7/18 |
| " | 32 | 1 | 3 and 12 | Further instructions by | 95 | | | 6/14/18 |
| " | 32 | | | Supplemented by | 111 | 8 | 2 | 7/8/18 |
| " | 32 | 3 | 1 | Revoked by | 152 | 1 | 3 | 9/10/18 |
| " | 32 | 1 | 2 | Amended by | 121 | 3 | 1 | 7/25/18 |
| " | 32 | 1 | 3 | Table given, revoked by | 121 | 3 | 2 | 7/25/18 |
| " | 33 | 4 | | If conflicting, revoked by | 73 | 3 | | 5/10/18 |
| " | 33 | 4 | | Revoked by | 147 | 3 | | 9/2/18 |
| " | 34 | 6 | | Revoked by | 131 | 1 | 1 | 8/7/18 |
| " | 35 | | | Army Corps School, amended by | 43 | 3 | | 3/2/18 |
| " | 35 | | 9 | Amended by | 77 | 6 | | 5/24/18 |
| " | 35 | | 5 | Amended by | 130 | 3 | 1 | 8/6/18 |
| " | 35 | | 13 | Revoked by | 133 | 6 | | 8/9/18 |
| " | 36 | | | Designation of Medical Officers, amended by | 88 | 1 | 2 | 6/6/18 |
| " | 38 | | | Designation of Medical Officers, amended by | 88 | 1 | 2 | 6/6/18 |
| " | 38 | 1 | | Supplemented by | 139 | 5 | | 8/24/18 |
| " | 40 | 1 | Sub. 8-A | Re reports of slightly wounded, revoked by | 77 | 8 | | 5/24/18 |

| Year. | G. O. No. | Section. | Paragraph. | REMARKS | G. O. No. | Section. | Paragraph. | Date. |
|---|---|---|---|---|---|---|---|---|
| 1918 | 40 | 1 | 8 | Re telegraphic reports of death, amended by | 100 | | 14 | 6/20/18 |
| " | 40 | 1 | 6 | Re making list of effects, amended by | 100 | | 14 | 6/20/18 |
| " | 40 | | 8-A | Amended by | 130 | 6 | 1 | 8/6/18 |
| " | 41 | 1 | 5 | Amended by | 88 | 3 | | 6/6/18 |
| " | 41 | 1 | 8 | Reports of Disability Boards, amended by | 100 | | 14 | 6/20/18 |
| " | 41 | | | Enlarged by | 161 | 1 | | 9/23/18 |
| " | 41 | 1 | | Supplemented by | 111 | 7 | 9 | 7/8/18 |
| " | 41 | | Art. 11 | Revoked by | 150 | 3 | 1 | 9/5/18 |
| | 43 | 4 | 4 | Extended by | 80 | 2 | | 5/28/18 |
| " | 43 | | | If conflicting, revoked by | 111 | 11 | 15 | 7/8/18 |
| " | 44 | | | Supplemented by | 102 | | 6 | 6/25/18 |
| " | 45 | 4 | | Amended by | 155 | 3 | | 9/13/18 |
| " | 45 | | | Supplemented by | 131 | 4 | 2 | 8/7/18 |
| " | 46 | | 4 and 7 | Re Service Records, where to be sent by | 100 | | 14 | 6/20/18 |
| " | 46 | | | Supplemented by | 105 | 9 | 2 | 6/28/18 |
| " | 46 | | | Revoked by | 111 | 1 | 1 | 7/8/18 |
| " | 48 | 4 | | Extended by | 83 | 2 | | 5/28/18 |
| " | 49 | | | Supplemented by | 102 | | 6 | 6/25/18 |
| " | 50 | | | Powers of R. R. and C. Service, extended by | 78 | 4 | 7 | 5/25/18 |
| " | 50 | 2 | | Reports of burial, supplemented by | 89 | 2 | | 6/7/18 |
| " | 50 | 3 | | Amended by | 157 | 1 | | 9/15/18 |
| " | 51 | 2 | 1 | If conflicting, revoked by | 116 | | 4 | 7/15/18 |
| " | 52 | 2 | | Revoked by | 80 | 1 | | 5/28/18 |
| " | 53 | 2 | | If conflicting, revoked by | 91 | 2 | | 6/10/18 |
| " | 54 | 2 | | Partly rescinded by | 152 | 5 | 2 | 9/10/18 |
| " | 56 | | | Explained by | 78 | 1 | | 5/25/18 |
| " | 56 | 2 | | Revoked by | 167 | 1 | | 10/1/18 |
| " | 65 | 3 | | Supplemented by | 102 | | 6 | 6/25/18 |
| " | 65 | 3 | | Amended by | 120 | 4 | | 7/24/18 |
| " | 66 | 1 | | If conflicting, amended by | 69 | 3 | | 5/5/18 |
| " | 68 | | 2 | Re Service Records, where to be sent by | 100 | | 14 | 6/20/18 |
| " | 68 | 1 | | Emphasized by | 165 | 2 | | 9/29/18 |
| " | 67 | | | Referring to "Table 25 - Ammunition Train", and "Summary" amended by | 82 | 1 | | 5/30/18 |
| " | 70 | | | Supplemented by | 144 | 6 | 1 | 8/29/18 |
| " | 72 | | | Partly amended by | 155 | 1 | 2 | 9/13/18 |
| " | 72 | | 9 | Amended by | 155 | 2 | | 9/13/18 |
| " | 73 | 3 | | Revoked by | 147 | 3 | | 9/2/18 |

| Year. | G. O. No. | Section. | Paragraph. | REMARKS | G. O. No. | Section. | Paragraph. | Date. |
|---|---|---|---|---|---|---|---|---|
| 1918 | 74 | | 2 | Re M. T. S., amended by | 114 | 1 | 3 | 7/11/18 |
| " | 78 | 3 | | If conflicting, revoked by | 146 | | | 9/1/18 |
| " | 79 | | 3 Sub. 5 | Supplemented by | 107 | | | 7/2/18 |
| " | 79 | 3 | 1 | Partly amended by | 93 | 1 | | 6/11/18 |
| " | 81 | 4 | 1 | Revoked by | 130 | 9 | 1 | 8/6/18 |
| " | 81 | 1 | | Supplemented by | 142 | 3 | | 8/27/18 |
| " | 81 | 3 | 5 | Revoked by | 157 | 2 | | 9/15/18 |
| " | 84 | | 2 | Partly revoked by | 135 | 3 | 3 | 8/17/18 |
| " | 89 | 1 | | Amended by | 130 | 4 | 1 | 8/6/18 |
| " | 91 | 2 | | Emphasized by | 158 | 4 | | 9/7/18 |
| " | 95 | | | Supplemented by | 121 | 3 | 3 | 7/25/18 |
| " | 99 | 2 | | Amended by | 105 | 4 | 1 | 6/28/18 |
| " | 100 | | 14 last Sub. Par. | Amended by | 130 | 6 | | 8/6/18 |
| " | 101 | | | If conflicting, revoked by | 146 | | | 9/1/18 |
| " | 102 | | 3-C | Referring to Col. A. T. Overshine, revoked by | 114 | 2 | | 7/11/18 |
| " | 102 | | 5-6-7 | Supplemented by | 115 | 2 | 3 | 7/12/18 |
| " | 104 | | (a) | Amended by | 159 | 2 | (a) | 9/19/18 |
| " | 105 | 1 | 3 | Amended by | 161 | 2 | | 9/23/18 |
| " | 106 | | | Supplemented by | 110 | 3 | 2 | 7/7/18 |
| " | 110 | | 4 | Amended by | 147 | 3 | | 9/2/18 |
| " | 111 | | | Supplemented by | 131 | 4 | 5 | 8/7/18 |
| " | 111 | | | Emphasized by | 165 | 2 | | 9/29/18 |
| " | 111 | 11 | | Explained by | 158 | 3 | | 9/17/18 |
| " | 116 | | | Amended by | 121 | 6 | | 7/25/18 |
| " | 118 | 1 | | Amended by | 155 | 4 | | 9/13/18 |
| " | 121 | 7 | | If conflicting, revoked by | 146 | | | 9/1/18 |
| " | 124 | | | Rescinded by | 162 | 1 | | 9/24/18 |
| " | 130 | 2 | 1 | Amended by | 159 | 3 | 1 | 9/19/18 |
| " | 131 | 2 | | Supplemented by | 135 | 3 and 6 | | 8/17/18 |
| " | 132 | 1 | 4 | Enlarged by | 167 | 3 | | 10/1/18 |
| " | 135 | 2 | | Amended by | 163 | 3 | | 9/25/18 |
| " | 139 | 4 | | Announcing Chief Veterinarian by | 142 | 6 | | 8/27/18 |
| " | 139 | 2 | | Revoked by | 157 | 3 | | 9/15/18 |
| " | 144 | 7 | | Amended by | 150 | 5 | 1 | 9/5/18 |
| " | 144 | 1 | | Rescinded by | 162 | 1 | | 9/24/18 |

### G. H. Q. BULLETINS

### AMERICAN EXPEDITIONARY FORCES

| Year. | G. O. No. | Bul. No. | Section. | Paragraph. | REMARKS | G. O. No. | Bul. No. | Section. | Paragraph. | Date. |
|---|---|---|---|---|---|---|---|---|---|---|
| 1917 | | 2 | 1 | | Supplemented by | 48 | | 1 | | 10/20/17 |
| " | | 3 | | | Revoked by | | 40 | 1 | | 6/22/18 |
| " | | 4 | 3 | | Amended by | | 60 | 1 | | 8/19/18 |
| " | | 10 | | | Supplemented by | 18 | | | | 1/31/18 |
| " | | 10 | | | Amplified by | 48 | | 3 | | 3/28/18 |
| " | | 10 | | | Amplified by | 50 | | 4 | | 3/30/18 |
| " | | 11 | 1 | | Explained by | | 16 | 1 | | 12 13/17 |
| " | | 11 | | | Explains | | 57 W.D. | | | 10/12/17 |
| " | | 11 | | | Re War Risk Insurance see | 5 | | | | 1/7/18 |
| " | | 11 | | | Re War Risk Insurance see | 25 | | 2 | | 2/9/18 |
| " | | 15 | | | Re War Risk Insurance see | 5 | | | | 1/7/18 |
| " | | 15 | | | Re War Risk Insurance see | 25 | | | | 2/9/18 |
| " | | 16 | | | Re War Risk Insurance see | 5 | | | | 1/7/18 |
| " | | 16 | | | Re War Risk Insurance see | 25 | | | | 2/9/18 |
| " | | 19 | | | Supplemented by | 44 | | | | 3/23/18 |
| " | | 20 | | 3 | Revoked by | | 20 | 1 | | 4/17/18 |
| " | 74 | | | 3-4 | Explained by | | 3 | 2 | | 1/15/17 |
| 1918 | | 6 | 1 | | Revoked by | | 15 | 1 | 1 | 3/6/18 |
| " | | 10 | | | If conflicting, revoked by | | 26 | | 9 | 5/11/18 |
| " | | 8 | | | Modified by | | 58 | 2 | | 8/15/18 |
| " | | 23 | | | Partly revoked by | | 42 | 2 | | 6/29/18 |
| " | | 25 | | | Supplemented by | | 56 | 1 | 1 | 8/13/18 |
| " | | 30 | | | Partly revoked by | 91 | | 1 | 5 | 6/10/18 |
| " | | 35 | 2 | 3-A | Amended by | | 48 | 1 | | 7/20/18 |
| " | | 60 | 1 | | Revoked by | | 66 | 2 | | 9/3/18 |

### SUBJECTS

| PURPORTS | G. O. | Bul. | Sec. | Par. |
|---|---|---|---|---|
| **Accidents** — | | | | |
| Caused by Government vehicles . . . . . | 29 | | III | 20 |
| **Accommodations** — | | | | |
| For disabled animals. . . . . . . . . . . . | 39 | | | (8b) |
| **Accountability** — | | | | |
| Animals. . . . . . . . . . . . . . . . . | 39 | | | (8a) |
| Of Officers . . . . . . . . . . . . . . . | 32 | | | 5 |
| Property-Supplies . . . . . . . . . . . . | 74 | | | |
| **Accounting** — | | | | |
| For Supplies . . . . . . . . . . . . . . | 43 | | | 10 |
| **Accounts** — | | | | |
| Auditing of . . . . . . . . . . . . . . . | | 18 | | |
| Money clothing, settlement of . . . . . . | 35 | | III | 1 |
| Of Q. M. Corps. . . . . . . . . . . . . | 31 | | III | |
| **Accuracy** — | | | | |
| Responsibility of Requisitions . . . . . . | 73 | | | 21 |
| **Acetylene Lamps** — | | | | |
| Authorized. . . . . . . . . . . . . . . . | 38 | | I | 7 |
| **Addresses** — | | | | |
| Field censorship regulations, rules governing (Rescinded in part by G. O. 48) . . | 13 | | II | 3 |
| Official, of organizations, men and officers of A. E. F. . . . . . . . . . . . | 11 | | | 2 |
| Procedure where addressee of letter not located . . . . . . . . . . . . . . . | 65 | | II | 3 |
| Procedure where addressee of letter unknown . . . . . . . . . . . . . . . | 65 | | II | 2 |
| Responsibility for proper, and officers and men informed of provisions of order . . | 11 | | | 2 |
| Specimen. . . . . . . . . . . . . . . . | 11 | | | 3 |
| **Adjustments** — | | | | |
| In pay directed. . . . . . . . . . . . . | 74 | | III | 4 |
| **Administrative** — | | | | |
| Function and command of French organization. . . . . . . . . . . . . . . . . | 40 | | II | 6 |
| **Administrative Section, G. S** . . . . . . . | 57 | | | |
| **Advance Depot, M. T. S.** . . . . . . . . . . | 70 | | | 7 |
| Departments comprising. . . . . . . . . | 75 | | | |
| Remount depots. . . . . . . . . . . . . | 39 | | | (4a) |
| Remount Service Personnel . . . . . . . | 39 | | | (4a) |
| Section, L. of C. . . . . . . . . . . . . | 20 | | III | 6 |
| **Advance Groups** — | | | | |
| Remount Service, L. of C. . . . . . . . | 39 | | | 4 |
| **Age Limits** — | | | | |
| Reserve Officers. . . . . . . . . . . . . | 54 | | | |

| PURPORTS | G. O. | Bul. | Sec. | Par. |
|---|---|---|---|---|
| **Alcohol, Solidified —** | | | | |
| Issue and instructions for use . . . . . . | 58 | | V | |
| Refilling empty cans for re-issue. . . . . | 58 | | V | 1 |
| Rules to secure results. . . . . . . . . . | 58 | | V | 2 |
| Shipment empty tins . . . . . . . . . | 58 | | V | 2 |
| **Alcoholic —** | | | | |
| Beverages forbidden. . . . . . . . . . . . | 77 | | | 12 |
| **Allotments —** | | | | |
| Compulsory and voluntary : | | | | |
| Enlistments prior to November 1st . . . | | 17 | | |
| General instructions. . . . . . . . . . . | 61 | | IV | |
| Pro-rating. . . . . . . . . . . . . . . | | 17 | | |
| Compulsory, War Insurance Bill . . . . | | 5 | I | 2 |
| Dead, discharged or deserted soldiers. . . | | 16 | II | |
| G. O. No 65, Sec. IV amended. . . . . . | 78 | | II | |
| Instructions in general, (amended by G. O. 78). . . . . . . . . . . . . . . . . | 65 | | IV | |
| Instructions, issue. . . . . . . . . . . . | | 5 | I | 1 |
| Officers. . . . . . . . . . . . . . . . . | 53 | | II | |
| Pay. . . . . . . . . . . . . . . . . . . | | 12 | | 1 |
| Of funds for purchase text books. . . . . | | 6 | I | 1-5 |
| Regular and Liberty Loan, abstracts. . . | 18 | | | |
| When paid . . . . . . . . . . . . . . . | 55 | | IV | 1 |
| **Allowances —** | | | | |
| Clothing, abolished after July 15, 1917. . | 16 | | | |
| Family . . . . . . . . . . . . . . . . . | | 12 | | 2 |
| Field, Shipment by “ grand vitesse ”. . . | 65 | | III | |
| Floor Space : | | | | |
| Enlisted men . . . . . . . . . . . . . | 46 | | I | 3-a |
| Hospitals . . . . . . . . . . . . . . . | 46 | | I | 3-d |
| Nurses . . . . . . . . . . . . . . . . | 46 | | I | 3-b |
| Officers. . . . . . . . . . . . . . . . | 46 | | I | 3-c |
| For clothing. . . . . . . . . . . . . . . | 16 | | | |
| Fuel : | | | | |
| For heating and cooking. . . . . . . . | 40 | | I | 2 |
| Wood and coal - tables of. . . . . . . | 40 | | I | 2 |
| Per diem : | | | | |
| Civilian employees in France . . . . . | 74 | | III | 3 |
| Commissioned officers and enlisted personnel. . . . . . . . . . . . . . . . | 76 | | III | |
| Of ∯2.00 to civilian employees and field clerks. . . . . . . . . . . . . . . . | 35 | | I | |
| Stove, amended . . . . . . . . . . . . . | 46 | | I | 5 |
| Tentage, officers. . . . . . . . . . . . . | 57 | | I | 3 |
| **American —** | | | | |
| Ambulance at Neuilly. . . . . . . . . . | 17 | | II | |
| Army. . . . . . . . . . . . . . . . . . | 7 | | | |
| Army mail . . . . . . . . . . . . . . . | 11 | | | |
| Citizens entering zone of A. E. F. . . . . | 29 | | II | 29 |
| Red Cross Bureau of Information and Casualties . . . . . . . . . . . . . . . . . | 81 | | II | 1 |
| Red Cross Mil. Hosp. No. 1 at Neuilly . . | 17 | | II | |
| Red Cross Mil. Hosp. No. 2 . . . . . . . | 51 | | II | |
| Red Cross Mil. Hosp. No. 3 . . . . . . . | 54 | | II | |
| Red Cross and Y.M.C.A. joint arrangements | 48 | | II | |

| PURPORTS | G. O. | Bul. | Sec. | Par. |
|---|---|---|---|---|
| **Ambulance —** | | | | |
| American at Neuilly, to be known as "American Red Cross Military Hospital No. 1" | 17 | | II | |
| Field, Service Depots, M. T. S. | 70 | | | 12 |
| **Amusements —** | | | | |
| Efforts to provide | 34 | | I | 2 |
| **Animals —** | | | | |
| Advance Veterinary Hospital | 39 | | | 4b |
| Captured, stray, issue, accountability for, reserve of, strength in numbers, delivery of, discharged from hospital, abandoned, collection | 39 | | | 8a |
| Disabled, discharged from hospital, care at ports of debarkation | 39 | | | 8b |
| Under treatment, details for care of | 42 | | I | 2 |
| **Anti-Gas Supplies —** | | | | |
| Distribution of | 53 | | I | 2 |
| **Appliances —** | | | | |
| And material for Gas Service | 31 | | IV | 4 |
| **Applications —** | | | | |
| For insurance to be completed and mailed to | 56 | | | |
| For insurance to be inspected | 56 | | | |
| For permits, will be in duplicate | 63 | | | 3 |
| For visits to Allied fronts | 63 | | II | 4 |
| Of civilian visitors and correspondents desiring to visit A. E. F. Headquarters or camps | 76 | | I | 2 |
| **Appointments and Promotions —** | | | | |
| Consideration Officers record | 76 | | II | 3 |
| First Corps | 64 | | | |
| Of Boards — fitness of officers | 62 | | | |
| Of Cadets to U. S. N. A. among enlisted men | 49 | | | |
| Recommendations | 76 | | II | 1 |
| Special Regulations | 76 | | II | 4 |
| Temporary | 76 | | II | 2 |
| **Arcachon** | 20 | | III | 3 |
| **Areas** | (20 | | | |
| Of L. O. C. Sections | (70 | | | |
| **Arctic Overshoes*** | 38 | | I | 1 |
| **Arm Bands —** | | | | |
| Description of | 59 | | | 1 |
| **Army —** | | | | |
| Center of information | 46 | | III | 1a |
| Exchanges prohibited | 33 | | III | 3 |
| Mobile Veterinary Hospital | 39 | | | 2 |
| M. T. S. Park, Organization of | 70 | | | 5 |
| Regulations, No. 803 modified | 38 | | III | |
| Remount Depot | 39 | | | 2 |
| Staff College | 46 | | III | 3 |
| Transport Service in Europe under D. G. T. | 78 | | I | |
| **Arrangements —** | | | | |
| For movements of incoming troops to be made by C. G. L. O. C. | 24 | | IV | 2 |
| Of billets | 46 | | I | 2 |

| PURPORTS | G. O. | Bul. | Sec. | Par. |
|---|---|---|---|---|
| **Arrest —** | | | | |
| Civilians where there is no A. P. M. . . . | 29 | | III | 19 |
| Non-commissioned officers. . . . . . . . | 29 | | III | 14-1 |
| Soldiers. . . . . . . . . . . . . . . . . | 29 | | III | 14 |
| **Articles —** | | | | |
| Sales, additional. . . . . . . . . . . . | 38 | | I | 2 |
| **Auditing of Accounts —** | | | | |
| Abstract of insurance premiums . . . . . | | 18 | | |
| Second Liberty Loan . . . . . . . . . . | | 18 | | |
| **Automatic —** | | | | |
| Supply — principle of. . . . . . . . . . | 43 | | | 6 |
| **Automobiles —** | | | | |
| Members Red Cross, Y. M. C. A., Salvation Army, and Knights of Columbus, travelling in. . . . . . . . . . . . . . . . . | 63 | | | 6 |
| Pass for chauffeurs of War Department. . | 63 | | | 6 |
| Regularly assigned officers. . . . . . . . | 63 | | | 6 |
| Passes. . . . . . . . . . . . . . . . . . | 63 | | | 6 |
| **Baggage —** | | | | |
| Report of unclaimed. . . . . . . . . . . | 60 | | IV | 2 |
| Stray or unclaimed . . . . . . . . . . . | 60 | | IV | 1 |
| Tracing lost. . . . . . . . . . . . . . | 71 | | | |
| **Base —** | | | | |
| Censor (Modified by G. O. 58, Sec. I, 1917). | 13 | | | |
| Groups, remount service. . . . . . . . . | 39 | | | 6 |
| Hospital unit (American Amb. at Neuilly). | 17 | | | |
| Remount depots. . . . . . . . . . . . . | 39 | | | 6a |
| Surgeon — charged with. . . . . . . . . | 77 | | | 4 |
| Veterinary hospital . . . . . . . . . . . | 39 | | | 6b |
| **Base Section No. 1** . . . . . . . . . . . . | 75 | | | |
| **Base Section No. 2** . . . . . . . . . . . . | 75 | | | |
| **Base Section No. 3** . . . . . . . . . . . . | 75 | | | |
| **Base Section No. 4** . . . . . . . . . . . . | 75 | | | |
| **Base Section No. 5** . . . . . . . . . . . . | 75 | | | |
| Agency for — troops arriving at channel ports . . . . . . . . . . . . . . . . . | 66 | | III | 1 |
| Duties in forwarding troops . . . . . . . | 66 | | III | 2 |
| Section office . . . . . . . . . . . . . | 66 | | III | |
| **Basis —** | | | | |
| Of automatic supply. . . . . . . . . . . | 73 | | | 28 |
| **Bath Houses —** | | | | |
| Authorized. . . . . . . . . . . . . . . . | 38 | | I | 2 |
| **Bedding —** | | | | |
| Straw for, furnished by Q. M. C. . . . . | 38 | | I | 16 |
| **Bellows Pockets —** | | | | |
| Will not be worn. . . . . . . . . . . . | 23 | | I | 2 |
| **Belt —** | | | | |
| Sam Browne and pistol, when worn . . | 23 | | I | 2 |
| U. S. Service . . . . . . . . . . . . . . | 18 | | III | |
| **Billeting —** | | | | |
| Faculty army schools . . . . . . . . . . | 46 | | III | 6 |
| Officers. . . . . . . . . . . . . . . . . | 57 | | I | 1 |
| Officers, men and other persons. . . . . | 46 | | I | 1 |
| Student officers Army Gen. Staff College. | 46 | | III | 6 |
| Troops . . . . . . . . . . . . . . . . . | | 10 | | |

| PURPORTS | G. O. | Bul. | Sec. | Par. |
|---|---|---|---|---|
| **Billets —** | | | | |
| Arrangement . . . . . . . . . . . . . . . | 46 | | I | 1 |
| Service of, etc. . . . . . . . . . . . . . | | 10 | | |
| **Benefits —** | | | | |
| Additional to War Risk Insurance . . . . | | 12 | | |
| Total disability . . . . . . . . . . . . . | | 14 | II | |
| **Boards —** | | | | |
| Appointing necessary for examination of officers . . . . . . . . . . . . . . . | 62 | | I | 2 |
| For reporting on suitability and fitness of officers . . . . . . . . . . . . . . . . | 62 | | I | 1 |
| Military, to examine and report capacity, etc., of officers . . . . . . . . . . . | 62 | | I | 2 |
| **Board, General Purchasing —** | | | | |
| Bringing order to attention of officers making requisition or purchases . . . . | 66 | | IV | 6 |
| Establishment of. . . . . . . . . . . . . | 23 | | II | 1 |
| Employing upon purchases persons paid by commission. . . . . . . . . . . . . | 53 | | IV | 1 |
| Formation of . . . . . . . . . . . . . . | 23 | | II | 2 |
| Meetings . . . . . . . . . . . . . . . . . | 23 | | II | 3 |
| Organization . . . . . . . . . . . . . . . | 28 | | | |
| Orders to purchasing agent . . . . . . . | 66 | | IV | 5 |
| Purchase of supplies or hire of civilians . | 23 | | II | 4 |
| Purchases not competing with other military interests . . . . . . . . . . . . . | 23 | | II | 3 |
| Purchasing and disbursing officers of Red Cross and Y.M.C.A. . . . . . . . . . | 23 | | II | 2 |
| Purchases subject to approval . . . . . . | 66 | | IV | 2 |
| Requisitions for material upon French Government made through. . . . . . . | 66 | | IV | 1 |
| Rules for handling small purchases at Paris and General Headquarters . . . . | 66 | | IV | 3 |
| Special branch to handle emergency and telegraphic requisitions . . . . . . . . | 66 | | IV | 4 |
| System of co-ordination of American purchases. . . . . . . . . . . . . . . . . | 53 | | IV | |
| **Board of Officers —** | | | | |
| (See Officers, Board of). | | | | |
| **Boots —** | | | | |
| That lace through entire lengths, when authorized. . . . . . . . . . . . . . . | 23 | | I | 2-3 |
| **Bordeaux**. . . . . . . . . . . . . . . . . . | 20 | | | 2b |
| **Bourges**. . . . . . . . . . . . . . . . . . . . | 20 | | IV | |
| **Building Material —** | | | | |
| Preventing misuse. . . . . . . . . . . . . | 51 | | IV | |
| **Bulletins —** | | | | |
| No. 7, voting in New York State, supply exhausted. | | | | |
| No. 8, publishes information on War Risk Insurance, supply exhausted. | | | | |
| No 2, Sec. 1, Supplemented by. . . . . . | 48 | | I | |
| No 11, Sec. 1, Explained by . . . . . . . | | 16 | I | |
| Publishing extracts . . . . . . . . . . . | 38 | | III | |
| To whom not distributed. . . . . . . . . | 38 | | III | |

| PURPORTS | G. O. | Bul. | Sec. | Par. |
|---|---|---|---|---|
| **Burial Department —** | | | | |
| And grave registration. . . . . . . . . . . | 27 | | II | b |
| Details of. . . . . . . . . . . . . . . . . | 27 | | IV | m |
| Establishment of. . . . . . . . . . . . . | 27 | | I | l |
| Ground - new . . . . . . . . . . . . . . . | 27 | | IV | k |
| Identification tag to accompany . . . . . | 21 | | | 6 |
| Places — requisition and maintenance . . | 27 | | II | h |
| Regulations pertaining to . . . . . . . . | 27 | | IV | |
| Burial to be reported . . . . . . . . . . | 27 | | IV | a |
| Burial to be under charge of. . . . . . . | 27 | | II | b |
| Under control of. . . . . . . . . . . . . | 27 | | III | |
| **Cablegrams —** | | | | |
| At reduced rates. . . . . . . . . . . . . | | 13 | II | 5 |
| Excessive number of personnel. . . . . . | 34 | | II | 1 |
| Indiscriminate source of danger . . . . . | 34 | | II | 2 |
| Instructions for preparation and accounting for tolls collected for transmission . . . | | 3 | | |
| Limited to absolutely necessary matters. . | 34 | | II | 3 |
| Rates, etc. . . . . . . . . . . . . . . . | 13 | | | |
| Restriction to a minimum . . . . . . . . | 34 | | II | 1 |
| Rules for . . . . . . . . . . . . . . . . | 42 | | III | 1-8 |
| Rules for preparation and transmittal. . . | | 3 | | |
| Rule No. 1. Persons entitled to privilege, character of contents. . . . . . . . . . | | 3 | I | a-b |
| Rule No. 2. Where messages may be filled in Europe and in the United States. . . | | 3 | II | a |
| Rule No. 3. Messages accepted subject delay and censorship. . . . . . . . . . . | | 3 | III | a-b |
| Rule No. 4. No minimum, but brevity desired, legibly written on proper forms. How to prepare messages. Plain English only permissible . . . . . . . . . | | 3 | IV | a-d |
| Rule No. 5. Importance address. How to count words in message . . . . . . . . | | 3 | V | a-b |
| Rule No. 6. Sender, surname required; full name and address recorded. . . . . . . | | 3 | VI | a |
| Rule No. 7. Charges prepaid in French money. No refunds . . . . . . . . . . | | 3 | VII | a-b |
| Rule No. 8. Duties receiving clerk, censorship, E.F.M. indication on envelopes. Receiver's register. Remittance . . . . . | | 3 | VIII | a-d |
| Rule No. 9. Accounting for tolls and settlement of bills . . . . . . . . . . . . | | 3 | IX | a-c |
| **Camps —** | | | | |
| Billeting troops in camp. . . . . . . . . | | 10 | | |
| Service of. . . . . . . . . . . . . . . . | | 10 | | |
| **Camp Sites —** | | | | |
| Occupied by Amexforce . . . . . . . . . | 7 | | | |
| **Canes —** | | | | |
| Not to be carried . . . . . . . . . . . . | 23 | | I | 2 |
| **Canned —** | | | | |
| Meats. Soups . . . . . . . . . . . . . . | 23 | | | 9 |
| **Card Index —** | | | | |
| Search of mail held at Headquarters for Staff Corps and Officers . . . . . . . . | 65 | | II | 4 |

| PURPORTS | G. O. | Bul. | Sec. | Par. |
|---|---|---|---|---|
| **Care** — | | | | |
| Of equipment | 43 | | | 10 |
| **Caring for Property** — | | | | |
| Public — Government | 74 | | II | |
| **Cars** — | | | | |
| Motor - markings | 60 | | | |
| To be placed by railway personnel | 73 | | | 20-e |
| To be loaded by depot personnel | 73 | | | 20 |
| (See Supplies, Cars). | | | | |
| **Cases** — | | | | |
| Approved for transfer to U.S. | 44 | | V | 4 |
| To be reported to A.P.M. | 29 | | III | 2 |
| Unfit for duty in France | 44<br>71 | | | |
| Cases of drunkenness subject to prompt disciplinary measures | 77 | | | 8 |
| **Casualties** — | | | | |
| Report of | 79 | | I | 6 |
| Bureau of Information of Casualties : | | | | |
| Information obtained by searchers | 81 | | II | 1 |
| Members allowed Medical and Hospital attention and privileges purchasing medicine. | 61 | | II | |
| Reports of searchers | 81 | | II | 2 |
| **Cemetery** | 27 | | IV | |
| **Censorship** — | | | | |
| Field Regulations (See Regulations Field Censorship). | | | | |
| Of cables | | 3 | | |
| Regimental | 13 | | II | 12 |
| Rules — Modification | 58 | | | |
| **Censor Stamps** — | | | | |
| Forwarding of correspondence, not properly stamped with, by postal agents at U.S.A.P.O.'s | 13 | | II | 13 |
| Issue of | 13 | | II | 21 |
| List of officers to whom will be issued | 13 | | Appx. | |
| On official envelopes and countersignature of officer | 22 | | III | |
| Stamping of mail of organizations not provided with | 13 | | II | 14 |
| **Certificates** — | | | | |
| Of Disability | 71 | | II | 1 |
| Of Disability not to be prepared in France. | 44 | | V | 4 |
| Required for issue of clothing | 16 | | | |
| **Changes** — | | | | |
| In supply list | 43 | | | 3 |
| **Chaplains** — | | | | |
| Co-operation of, with Red Cross and Y.M.C.A | 26 | | II | 3 |
| Functions of | 26 | | II | 5 |
| Not to call upon Red Cross or Y.M.C.A. to perform functions of other, except proper agency is unable to accomplish desired results | 26 | | II | 3 |
| To acquaint themselves | 27 | | IV | f |

| PURPORTS | G. O. | Bul. | Sec. | Par. |
|---|---|---|---|---|
| **Circulation —** | | | | |
| Identification - personnel A. E. F. . . . . | 63 | | | |
| **Cities —** | | | | |
| In France, having the same name, to be clearly defined. . . . . . . . . . . . . | 41 | | III | |
| **Civilians —** | | | | |
| Accurate and complete records; statistical division for . . . . . . . . . . . . . . | 67 | | I | 1-2 |
| Arrested where there is no A. P. M. . . | 29 | | III | 19 |
| Co-operation with officers and soldiers to insure temperance and prevent ravages venereal diseases . . . . . . . . . . . | 34 | | I | 1 |
| Disposition of dismissed or discharged . . | 22 | | II | |
| Employees hired locally to be reported. . | 24 | | II | |
| Free postage provision. . . . . . . . . . | 13 | | III | |
| To whom applying . . . . . . . . . . | 54 | | III | 1 |
| To whom not applying. . . . . . . . . | 68 | | I | 1 |
| General Order No. 54, so much of Sec. 3 as relates to civilians, revoked . . . . . . | 68 | | I | 2 |
| Identification tags. . . . . . . . . . . . | 21 | | VI | |
| Shipment by " Grande vitesse "; field allowance. . . . . . . . . . . . . . . | 65 | | III | |
| Visitors, regulations governing. . . . . . | 76 | | I | |
| **Civilian Employees** | | | | |
| Alphabetical list. . . . . . . . . . . . . | 2<br>25 | | I<br>IV | |
| Amusements, etc., when off duty. . . . . | 34 | | I | 2 |
| Discussing military information. . . . . . | 10 | | II | |
| Mail addressed to deceased. . . . . . . . | 44 | | III | |
| Pay : | | | | |
| Adjustments in . . . . . . . . . . . . | 74 | | III | 4 |
| Classes " A " to " D ". . . . . . . . . . | 74 | | III | 2 |
| Per diem, allowance of $2.00 : | | | | |
| When entitled to . . . . . . . . . . . | 35 | | II | |
| When not entitled to . . . . . . . . . | 35 | | II | 1 |
| Per diem, allowances or expenses . . . . | 74 | | III | 3 |
| Permanent, allotments. . . . . . . . . . | | 5 | I | 1 |
| Photographs. . . . . . . . . . . . . . . | 85 | | | 4 |
| Registering after arrival in Paris . . . . | 30 | | | 3 |
| Visiting French and British Army Zone. . | 63 | | | 4 |
| " Workers' Permits ", General instructions. | 63 | | | 3 |
| **Civilian Laborers —** | | | | |
| Employment of, together with American and French soldiers on non-military work | 48 | | III | 1 |
| Personnel, where procured and wages paid. | 48 | | III | 2 |
| **Civil Post Office —** | | | | |
| Use of, prohibited. . . . . . . . . . . | 13 | | | |
| **Civil Telegraph**. . . . . . . . . . . . . . . | 13 | | II | 24 |
| **Classification —** | | | | |
| And distribution of supplies . . . . . . . | 73 | | | 16 |
| Of civilian employees . . . . . . . . . . | 74 | | III | 1 |
| **Clothing —** | | | | |
| Allowance for. . . . . . . . . . . . . . | 16 | | | |
| Articles of, other than uniform pattern, wearing of . . . . . . . . . . . . . . | 23 | | I | 3 |

| PURPORTS | G.O. | Bul. | Sec. | Par. |
|---|---|---|---|---|
| **Clothing —** | | | | |
| Clothing allowance abolished after July 15. | 16 | | | |
| Determination of unserviceability. . . . . | 23 | | I | 3 |
| French Interpreters : | | | | |
| Clothing worn by. . . . . . . . . . . | 46 | | IV | 2 |
| Issue of clothing to . . . . . . . . . . | 46<br>41 | | IV<br>V | 1<br>4 |
| Lost or destroyed charged on payrolls . . | 16 | | | |
| Purchase by employees of P. O. Dept. . . | 15 | | II | |
| Salvage of, renovating, repairing and re-issuing . . . . . . . . . . . . . . . . | 16 | | | |
| Shops for repair of . . . . . . . . . . . | 23 | | I | 3-3 |
| To be cleaned, disinfected, etc., salvage depot | 68 | | IV | 4 |
| Worn out or damaged to be turned in to Q. M. C . . . . . . . . . . . . . . . . | 16 | | | |
| Small articles sent as gifts, mailing of . . | 58 | | I | b |
| **Coat —** | | | | |
| And hat, wearing of . . . . . . . . . . | 23 | | I | 2 |
| **Commissaries, Sales** . . . . . . . . . . . . | 38 | | I | 12 |
| **Communications —** | | | | |
| Line of, (See Line of Communications). | | | | |
| With French authorities, preparation of. | 40 | | II | 7-a-e |
| With French Military Commission . . . . | 40 | | II | 3 |
| **Commutation of Rations —** | | | | |
| Patients of hospitals, vouchers for. . . . | 24 | | I | |
| When paid at the rate of $2.00 per day : | | | | |
| Enlisted chauffeurs . . . . . . . . . . | 42 | | II | 1 |
| Soldiers. . . . . . . . . . . . . . . . . . | 42 | | II | 2 |
| **Commutation, Officers, Quarters —**. . . . . | 57 | | I | 4 |
| **Company Control of Mail**. . . . . . . . . . | 13 | | II | 11 |
| **Conduct —** | | | | |
| Officers and men . . . . . . . . . . . . | 32 | | III | |
| On arrival of American officers or troops in French towns or stations . . . . . . | 59 | | II | 1 |
| Transaction of business with Allies. . . . | 59 | | II | |
| **Confidential Matters —** | | | | |
| Care to be taken in treatment . . . . . . | 64 | | I | 1 |
| Considered " Official ". . . . . . . . . . | 64 | | I | 2 |
| How prepared for publication . . . . . . | 64 | | I | 4 |
| Kept under lock and key . . . . . . . . | 64 | | I | 3 |
| Marking. . . . . . . . . . . . . . . . . | 64 | | I | 5 |
| Signification of word " Secret " on communications . . . . . . . . . . . . . . | 64 | | I | 3 |
| **Contagious Diseases**. (See **Diseases**.) | | | | |
| **Conversations —** | | | | |
| Relative to military information. . . . . | 10 | | II | 2 |
| **Cookers —** | | | | |
| Fireless. . . . . . . . . . . . . . . . . . | | 10 | | |
| **Cooking —** | | | | |
| Allowances of fuel wood for. . . . . . . | 40 | | I | 2 |
| Wood for . . . . . . . . . . . . . . . . | | 10 | | |
| **Correspondence —** | | | | |
| Copies of, authorized by Par. 3 and 4, Sec. 2, G. O. 40 No. . . . . . . . . . . . . | 57 | | II | |
| Detail information of assignments. . . . | 33 | | II | 3 |

| PURPORTS | G. O. | Bul. | Sec. | Par. |
|---|---|---|---|---|
| **Correspondence —** | | | | |
| Forwarding of, not properly stamped with censor stamp, by postal agents of U. S. A. Post Offices, forbidden . . . . . . . . . | 13 | | II | 13 |
| Forwarding without prepayment of postage. . . . . . . . . . . . . . . . . . . | 13 | | II | 13 |
| Names of officers typewritten on. . . . . | 44 | | IV | |
| Relative to shipment from base thru Hqs. L. O. C. . . . . . . . . . . . . . . . . | 26 | | I | 4 |
| With strangers, etc. . . . . . . . . . . | 13 | | II | 5 |
| With prisoners of war. . . . . . . . . . | 13 | | II | 6 |
| **Correspondence with French Authorities —** | | | | |
| Communications, preparation of . . . . . | 40 | | II | 7-a-e |
| French Military Mission : | | | | |
| Act for French Minister of War . . . . | 40 | | II | 3 |
| Communication with . . . . . . . . . | 40 | | II | 3 |
| Letters to be signed "By Direction". . | 40 | | II | 3 |
| General character . . . . . . . . . . . . | 40 | | II | 1 |
| Letters of certain classes signed by C. of S. | 40 | | II | 4 |
| Liaison officer, special. . . . . . . . . . | 40 | | II | 2 |
| Liaison officers, use of. . . . . . . . . . | 40 | | II | 5 |
| Memorandum "Correspondence between French and American Military Authorities" revoked . . . . . . . . . . . . | 40 | | II | 8 |
| Rules relating to correspondence, liaison, etc. . . . . . . . . . . . . . . . . | 40 | | II | 6 |
| **Corps,** 1st **Center of Instruction —** | | | | |
| Established . . . . . . . . . . . . . . . . | 45 | | I | |
| Organization : | | | | |
| Field Officers; school; director . . . . | 45 | | IV | h |
| 1st Army Corps Schools, commandants. | 45 | | IV | |
| Gas school; director. . . . . . . . . . | 45 | | IV | d |
| Instructors . . . . . . . . . . . . . . . | 45 | | IV | i |
| Sanitary school; director. . . . . . . . | 45 | | IV | f |
| Troops . . . . . . . . . . . . . . . . . . | 45 | | IV | last |
| **Corps —** | | | | |
| Medical, Director Laboratories . . . . . . | 58 | | IV | |
| Medical, Reserve : | | | | |
| Director, Division of Gen. Surgery. . . | 58 | | IV | |
| Director, Orthopedic Surgery . . . . . | 58 | | IV | |
| Director, Venereal, Skin and G-U Surgery . . . . . . . . . . . . . . . . | 58 | | IV | |
| Physical examination of officers by Medical. . . . . . . . . . . . . . . . . . . | 67 | | IV | 1 |
| **Corps, Marine —** | | | | |
| Part of Regular Army . . . . . . . . . . | 31 | | III | |
| Supplies furnished, accounting for. . . . | 31 | | III | 1 |
| **Corps, Officers Reserve —** | | | | |
| Discharging soldiers receiving commission in . . . . . . . . . . . . . . . . . . . . | 63 | | V | 2 |
| **Court Martials —** | | | | |
| For contracting venereal disease . . . . . | 6 | | | |
| **Court Martials, General —** | | | | |
| Record of trial by, to whom forwarded. . | 19 | | IV | 1 |
| **Covers, Horse** . . . . . . . . . . . . . . | 38 | | I | 5 |

| PURPORTS | G. O. | Bul. | Sec. | Par. |
|---|---|---|---|---|
| **Damages** — | | | | |
| Claims, report of . . . . . . . . . . | 29 | | III | 25 |
| To private property . . . . . . . . . . . | 7 | | | |
| **Deaths** — | | | | |
| Of soldiers having allotments or War Risk Insurance . . . . . . . . . . . . . . . . | | 16 | II | |
| Report of, of officers. . . . . . . . . . . | 19 | | I | |
| **Definitions** — | | | | |
| Term " Commissioned Officer ". . . . . . . | | 11 | | |
| Terms in War Risk Insurance Act . . . . | | 12 | | |
| **Departments**, A. E. F. . . . . . . . . . . . | | | | |
| Heads. . . . . . . . . . . . . . . . . . . . | 1 | | | |
| **Department** — | | | | |
| Members American Army . . . . . . . . | 7 | | | |
| **Depots** — | | | | |
| Remount Service : | | | | |
| Advance remount . . . . . . . . . . . | 39 | | IV | a |
| Base remount . . . . . . . . . . . . . | 39 | | VI | a |
| Intermediate remount . . . . . . . . . | 39 | | V | a |
| Remount . . . . . . . . . . . . . . . . | 39 | | VIII | a |
| **Depredations Made in Billets** . . . . . . . | | 10 | | |
| **Diaries, War** — | | | | |
| Number copies and where forwarded. . . | 2 | | V | |
| **Disbursing Officers, Temporary** — | | | | |
| Transfer of balances. . . . . . . . . . . | 19 | | II | |
| **Diseases** —. . . . . . . . . . . . . . . . . | | | | |
| Contagious, observation, instruction . . . | 84 | | III | |
| Typhoid fever, prophylaxis against. . . . | 84 | | II | |
| **Diseases, Venereal** — | | | | |
| Advice. . . . . . . . . . . . . . . . . . . | 6 | | | |
| Charge preventive regulations . . . . . . | 77 | | IV | |
| Dangers. . . . . . . . . . . . . . . . . . | 34 | | I | 2(2) |
| Effectives in various commands. . . . . . | 77 | | 13 | |
| Effects of contracting . . . . . . . . . . | 6 | | | |
| Evil effects . . . . . . . . . . . . . . . | 6 | | | |
| Infirmaries for treatment. . . . . . . . . | 34 | | I | 4 |
| List of men suffering from . . . . . . . | 77 | | IX | |
| Literature on subject. . . . . . . . . . . | 77 | | 14 | |
| Minimizing . . . . . . . . . . . . . . . . | 6 | | | |
| " Off Limits ". . . . . . . . . . . . . . | 77 | | 3 and 12 | |
| Orders relating to : | | | | |
| Accountability for compliance . . . . . | 77 | | last | |
| Application . . . . . . . . . . . . . . | 77 | | XV | |
| Passes to transient troops . . . . . . . . | 77 | | VI | |
| Posting G. O. No. 6 relating to . . . . . | 6 | | | |
| Prevalance and dangers . . . . . . . . . | 34 | | I | 3 |
| Preventing ravages . . . . . . . . . . . | 34 | | I | 1 |
| Prevention . . . . . . . . . . . . . . . . | 77 | | II | |
| Preventive measures. . . . . . . . . . . | 34 | | I | 2-(2) |
| Prophylactic Stations : | | | | |
| Reported to, when exposed to venereal diseases. . . . . . . . . . . . . . . . | 77<br>6 | | VII | |
| Treatment at, of men returning intoxicated condition . . . . . . . . . . . . . . | 77 | | VIII | |
| Where established. . . . . . . . . . . | 6 | | | |

| PURPORTS | G. O. | Bul. | Sec. | Par. |
|---|---|---|---|---|
| **Diseases, Venereal —** | | | | |
| Protection from | 6<br>34 | | I | 8 |
| Punishment for contracting | 6<br>34 | | I | 7 |
| Report on number of cases | 77 | | X | |
| Report physical condition troops | 77 | | V | |
| Special hospitals | 34 | | I | 6 |
| Supplementary instruction | 77 | | I | |
| Treatment | 34 | | I | 5 |
| Trial by court martial for contracting | 6 | | | |
| **Discipline and Efficiency —** | | | | |
| Maximum of officers and men | 32 | | II | |
| **Discussion —** | | | | |
| Of military information prohibited except in line of duty | 10 | | II | |
| **Doctors —** | | | | |
| Free postage provision | 54 | | III | 1 |
| **Documents —** | | | | |
| Confidential | 64 | | I | 4b |
| For ordinary circulation | 64 | | I | 4a |
| Marking | 64 | | I | 5 |
| Secret | 64 | | I | 4c |
| **Economy —** | | | | |
| In use of fuel wood, coal, gasoline, lubricating oils, fuel oil and greases | 40 | | I | 1 |
| **Employees, Civilian —** | | | | |
| Alphabetical list | 2<br>25 | | I<br>IV | |
| Amusements, etc., when off duty | 34 | | I | 2 |
| Discussing military information | 10 | | II | |
| Mail addressed to deceased | 44 | | III | |
| Pay : | | | | |
| Adjustments in | 74 | | III | 4 |
| Classes "A" to "D" | 74 | | III | 2 |
| Per diem, allowance of $2.00 : | | | | |
| When entitled to | 35 | | II | |
| When not entitled to | 35 | | II | |
| Per diem, allowances and expenses | 74 | | III | 3 |
| Permanent, allotments | | 5 | I | 1 |
| Photographs | 85 | | | 4 |
| Registering after arrival in Paris | 30 | | | 3 |
| Visiting French or British army zones | 63 | | | 4 |
| "Workers Permits", General instructions | 63 | | | 3 |
| **Employment —** | | | | |
| Of American and French soldiers together with civilian laborers on non-military work | 48 | | III | 1 |
| Of civilians residing in Europe | 13 | | III | |
| **Enlisted Men —** | | | | |
| Accurate and complete records, Statistical division for | 67 | | I | 1-2 |
| Allotments | 65<br>78 | | IV<br>II | |
| Allotments, compulsory | | 5 | I | 2 |
| Allowance, floor space | 46 | | I | 3-a |

| PURPORTS | G. O. | Bul. | Sec. | Par. |
|---|---|---|---|---|
| **Enlisted men** (Cont'd) — | | | | |
| Alphabetical list | 2<br>25 | | I<br>IV | |
| Amusements, etc., when off duty | 34 | | I | 2 |
| Appointment as cadets in U. S. Mil. Academy | 49 | | | |
| Arrest of | 29 | | III | 14 |
| Artillery, insignia | | 20 | II | |
| Assigment of, experienced in handling mail. | 65 | | II | 1 |
| Billeting | 46 | | I | 1 |
| Carried on separate muster rolls | 68 | | II | |
| Commutation, rations paid at rate of $ 2.00 per day | 42 | | II | 2 |
| Compulsory and voluntary allotments : General instructions | 61 | | IV | |
| Conduct | 32 | | III | |
| Co-operation with officers and civilians to insure temperance and prevent ravages of venereal diseases | 34 | | I | 1 |
| Death, discharge, desertion, having allotments or War Risk Insurance | | 18 | II | |
| Deportment and treatment, French people. | 7 | | | |
| Deposits, abstracts | | 18 | | |
| Determination, patriotism, loyalty and efficiency | 32 | | | |
| Discharged in France | 44 | | I | |
| Discharged on certificate of disability | 44 | | I | 1 |
| Discharging those receiving commissions in Federal Service | 68 | | III | 1 |
| Discharging those receiving commissions in Officers' Reserve Corps | 68 | | III | 2 |
| Disposition of dismissed and discharged | 22 | | II | |
| Discussing military information | 10 | | II | |
| Employment of American and French together on non-military work | 48 | | III | |
| Entitled to mail letters free | 48 | | I | |
| Free entry of parcels sent to | | 1 | I | |
| Free postage provision | 54 | | III | 1 |
| Identity card | 63 | | I | |
| Identification tags | 21 | | VI | |
| Mail addressed to deceased | 44 | | III | |
| Punishment for contracting venereal diseases | 34<br>6 | | I | 7 |
| Money, clothing accounts, settlement of | 35 | | III | 1 |
| Per diem allowance | 76 | | III | |
| Official address | 10 | | III | |
| Registered at hotels and pensions, list of. | 29 | | III | 5 |
| Regular Army, National Guard and National Army on separate muster roll. | | | | |
| Registering after arrival in Paris | 30 | | I | 3 |
| Reporting at A. P. M. Offfice | 29 | | III | 2 |
| Report of death, discharge or desertion | 19 | | I | |
| Saluting, meaning and importance | 23 | | I | 5 |
| Shipment field allowance by " Grande Vitesse " | 65 | | III | |

| PURPORTS | G. O. | Bul. | Sec. | Par. |
|---|---|---|---|---|
| **Enlisted men** (Cont'd) — | | | | |
| Travel by rail, 3rd Class . . . . . . . . | 64 | | II | 1 |
| Under arrest, forwarding charges, etc. . . | 29 | | III | 7 |
| Visiting French or British army zones . . | 63 | | IV | |
| Without pass or overstaying pass. . . . . | 29 | | III | 2 |
| Written permission to visit Paris. . . . . | 30 | | I | 2 |
| **Entry of Parcels** — | | | | |
| Free to officers and men of A. E. F. . . . | | 1 | I | |
| **Envelopes** — | | | | |
| Blue . . . . . . . . . . . . . . . . . . . . | 13 | | II | 10 |
| Countersignature of officer and censor stamp on official . . . . . . . . . . . | 22 | | III | |
| **Examination, Physical** — | | | | |
| Conducted by 2 officers, Medical Corps. . | 67 | | IV | |
| Officers. . . . . . . . . . . . . . . . . . | 67 | | IV | |
| **Floor space** — | | | | |
| Allowance of, (See **Allowances**). | | | | |
| **Franking Privilege** — | | | | |
| Over French telegraphic lines . . . . . . . | 21 | | V | |
| **French military Mission** — | | | | |
| Act for French Minister of War . . . . . | 40 | | II | 3 |
| Communication with . . . . . . . . . . | 40 | | II | 3 |
| Letters to be signed " by Direction ". . . | 40 | | II | 3 |
| **Front** — | | | | |
| Officers vicinity French or British . . . . | 19 | | III | |
| **Fuel Oil** — | | | | |
| Economy in use. . . . . . . . . . . . . . | 40 | | I | 1 |
| Intermediary between A. E. F. and French Gov't concerning supply. . . . . . . . | 40 | | I | 3 |
| Requisition . . . . . . . . . . . . . . . . | 40 | | I | 4 |
| **Fuel Wood** — | | | | |
| Allotment to French soldiers. . . . . . . | 40 | | I | 2 |
| Allowance of, for heating and cooking . . | 40 | | I | 2 |
| Economy in use . . . . . . . . . . . . | 40 | | I | 1 |
| Intermediary between A. E. F. and French Government concerning supply. . . . . | 40 | | I | 3 |
| Quantity . . . . . . . . . . . . . . . . . | 40 | | I | 3 |
| Requisitions. . . . . . . . . . . . . . . . | 40 | | I | 4 |
| **Gas Masks** — | | | | |
| Issue of. . . . . . . . . . . . . . . . . . | 53 | | I | 2 |
| **Gas service** — | | | | |
| Alarm devices, how treated and issued. . | 53 | | I | 3 |
| All anti-gas supplies furnished by . . . . | 53 | | I | 1 |
| Chief of, and duties. . . . . . . . . . . | 31 | | IV | 2 |
| Establishment of . . . . . . . . . . . . . | 31 | | IV | 1 |
| Issue of masks and respirators . . . . . . | 53 | | I | 2 |
| Material and appliances . . . . . . . . . | 31 | | IV | 4 |
| Material, supply of . . . . . . . . . . . . | 31 | | IV | 5 |
| Organization per table attached . . . . . | 31 | | IV | 3 |
| **Gasoline** — | | | | |
| Economy in use. . . . . . . . . . . . . | 40 | | I | 1 |
| Intermediary between A. E. F. and French Gov't concerning supply. . . . . . . . | 40 | | I | 3 |
| Requisitions. . . . . . . . . . . . . . . | 40 | | I | 4 |

| PURPORTS | G.O. | Bul. | Sec. | Par. |
|---|---|---|---|---|
| **General orders —** | | | | |
| Modifying 803 A. R. . . . . . . . . . . . | 38 | | III | |
| No. 2, Par. 2 Amended . . . . . . . . . | 24 | | III | |
| No. 2, Amended. . . . . . . . . . . . . | 67 | | I | 9-10 |
| No. 3 and 5, Revoked . . . . . . . . . | 13 | | I | |
| No. 13, Distribution to troops upon arrival in France . . . . . . . . . . . . . . . | 13 | | II | 31 |
| No. 13, Modified. . . . . . . . . . . . | 58 | | I | a-d |
| No. 13, Rescinded in part . . . . . . . . | 48 | | I | |
| No 14, Withdrawn, as it published a table of organization now obsolete. | | | | |
| No. 13, Violations of. . . . . . . . . . . | | 2 | I | |
| No. 18, Par. 3, Revoked . . . . . . . . . | 41 | | IV | |
| No. 19, Par. 1, Amended . . . . . . . . | 67 | | I | 9 |
| No. 20, Sec. III, Par. 4, Amended . . . . | 66 | | III | 1-3 |
| No. 20, Amended . . . . . . . . . . . . | 75 | | | |
| No. 26, Par. 2, amplified. . . . . . . . . | 48 | | II | |
| No. 41, Sec. IV, Par. 5, Revoked. . . . . | 80 | | II | 1 |
| No. 44, Sec. V, Par. 4, Amended. . . . . | 71 | | II | 1 |
| No. 46, Sec. II, Revoked. . . . . . . . . | 80 | | I | 4 |
| No. 52, List of articles of rations for Thanksgiving (Supply exhausted). | | | | |
| No. 54, Par. 3, Amended in part . . . . . | 68 | | I | 2 |
| No. 65, Sec. IV, Amended. . . . . . . . | 78 | | II | |
| No. 67. Sec. III, Amended . . . . . . . . | 78 | | III | |
| No. 68, Sec. III, Amended. . . . . . . . | 75 | | | |
| No. 69, List of articles of rations for Thanksgiving (Supply exhausted. | | | | |
| No. 72, Announces opportunity to participate in 3rd French Loan (Supply now exhausted). | | | | |
| Officers not relieved of certain duties by No. 67. . . . . . . . . . . . . . . . | 67 | | I | 8 |
| Procedure to carry into effect provisions of No. 89 W. D. (General Order No. 16, A. E. F.) . . . . . . . . . . . . . . . | 35 | | III | |
| Publishes list of articles of rations for Christmas Day (Supply now exhausted). | 69 | | | |
| Publishing extracts . . . . . . . . . . . | 38 | | III | |
| To whom distributed. . . . . . . . . . . | 38 | | III | |
| Violation of No. 13 Re : Postal Regulations. . . . . . . . . . . . . . . . . | | 2 | I | |
| **General Purchasing Board —** | | | | |
| Organization . . . . . . . . . . . . . . | 28 | | | |
| **Gifts —** | | | | |
| Gloves sent as, mailing of. . . . . . . . | 58 | | I | p |
| Letters from strangers, acknowledgement. | 13 | | II | |
| **Gloves —** | | | | |
| Leather or mittens, use authorized . . . . | 38 | | I | [illegible] |
| Sent as gifts, mailing of. . . . . . . . . | 58 | | I | d |
| **Groups —** | | | | |
| Remount. Service : | | | | |
| Advance . . . . . . . . . . . . . . . | 39 | | IV | |
| Base . . . . . . . . . . . . . . . . . | 39 | | VI | |
| Intermediate . . . . . . . . . . . . . | 39 | | V | |

| PURPORTS | G. O. | Bul. | Sec. | Par. |
|---|---|---|---|---|
| **Hat and Coat** — | | | | |
| Service, wearing of . . . . . . . . . . . . | 23 | | I | 2 |
| **Hospitals** — | | | | |
| Allowance, floor space. . . . . . . . . . . | 46 | | I | 3d |
| American ambulance at Neuilly to be known as "American Red Cross Mil. Hospital No. 1" . . . . . . . . . . . . . . . . . | 17 | | | |
| American Red Cross : | | | | |
| Control . . . . . . . . . . . . . . . . . | 51 | | II | 1 |
| Name. . . . . . . . . . . . . . . . . . . | 51 | | II | 1 |
| Taking over. . . . . . . . . . . . . . . | 51 | | II | |
| L'Hopital Auxiliaire No. 53 : | | | | |
| Control . . . . . . . . . . . . . . . . . | 54 | | II | 1 |
| Name . . . . . . . . . . . . . . . . . . | 54 | | II | 1 |
| Taking over. . . . . . . . . . . . . . | 54 | | II | |
| Special, for treatment of venereal diseases. | 34 | | I | 6 |
| U. S. A. activities of Y. M. C. A. and Am. Red Cross in . . . . . . . . . . . . . . | 48 | | II | |
| Voucher for commutation of rations. . . . | 24 | | I | 1 |
| **Hospitals, Veterinary** — | | | | |
| Remount Service : | | | | |
| Accommodation for disabled animals . . | 39 | | VIII | b |
| Advance, personnel . . . . . . . . . . . | 39 | | IV | b |
| Base, personnel . . . . . . . . . . . . . | 39 | | VI | b |
| Intermediate . . . . . . . . . . . . . . | 39 | | V | b |
| Mobile and stationary . . . . . . . . . . | 39 | | I | |
| **Identification Tags** — | | | | |
| Disposition of, on death of wearer . . . . | 21 | | VI | 3 |
| Enforcement of order . . . . . . . . . . . | 21 | | VI | 2 |
| Second aluminum tag . . . . . . . . . . . | 21 | | VI | 1 |
| To be worn by civilians attached to A. E. F. . . . . . . . . . . . . . . . . . . | 21 | | VI | |
| Violations of order . . . . . . . . . . . . | 21 | | VI | 2 |
| Wearing of two tags . . . . . . . . . . . | 21 | | VI | 2 |
| **Identity Cards** — | | | | |
| Application for, to supply officers . . . . | 63 | | I | |
| Carrying by officers. . . . . . . . . . . . | 63<br>85 | | I<br>I | |
| Furnishing soldiers leaving divisional area, specimen . . . . . . . . . . . . . . . . | 63 | | II | |
| Right to railway warrant or ticket for travel by rail . . . . . . . . . . . . . . | 63 | | VII | |
| Stamping and signing . . . . . . . . . . . | 63 | | I | |
| "Workers' Permit" : | | | | |
| Carrying of same . . . . . . . . . . . . | 85 | | I | |
| General instructions. . . . . . . . . . . | 63 | | III | |
| **Infirmaries** — | | | | |
| For treatment of venereal diseases . . . . | 34 | | I | 4 |
| **Instruction, 1st Corps, Centre of** (See **Corps — 1st. Center of Instruction**). | | | | |
| **Instructions** — | | | | |
| As to disposition of sick and wounded (Amended by G. O. 71) . . . . . . . . . | 44 | | V | 1-4 |
| For guidance of Military Police . . . . . | 29 | | | |
| Prevention venereal diseases. . . . . . . | 77 | | I | |

| PURPORTS | G. O. | Bul. | Sec. | Par. |
|---|---|---|---|---|
| **Insurance, War risk** — | | | | |
| (See **War Risk Insurance**) — | | | | |
| **Intemperance** — | | | | |
| Alcoholic beverages, buying, gifts. . . . | 77 | | | 12 |
| Effectives in various commands. . . . . . | 77 | | | 13 |
| Limiting places where intoxicants sold. . | 77 | | | 11 |
| Literature on subject. . . . . . . . . . . | 77 | | | 14 |
| Orders relating to : | | | | |
| Accountability for compliance . . . . . | 77 | | | last |
| Application . . . . . . . . . . . . . . | 77 | | | 15 |
| **Killed** — | | | | |
| Reports of. . . . . . . . . . . . . . . . | 2 | | III | |
| **Kitchens** — | | | | |
| Rolling . . . . . . . . . . . . . . . . . | 38 | | I | 10 |
| **Laborers, Civilian** — | | | | |
| Employment of, together with American and French Soldiers on non-military work . . . . . . . . . . . . . . . . . . | 48 | | III | 1 |
| Personnel, where procured and wages paid. . . . . . . . . . . . . . . . . . | 48 | | III | 2 |
| **Latrines** — | | | | |
| Havard boxes and oil not obtainable for . | 12 | | I | |
| Instructions for construction of. . . . . . | 12 | | II | |
| Plan of . . . . . . . . . . . . . . . . . | 12 | | II | |
| Present approved type satisfactory; disinfection . . . . . . . . . . . . . . . . | 38 | | I | 6 |
| **Letters** — | | | | |
| Civilians . . . . . . . . . . . . . . . . | 13 | | II | 17 |
| Free postage provision. . . . . . . . . . | 54 | | III | 1 |
| No postage required for certain. . . . . . | 15 | | I | |
| Of certain classes signed by C. of S. . . . | 40 | | II | 4 |
| Of gifts from strangers, acknowledgment. | 13 | | II | 7 |
| Soldiers, sailors and marines, entitled to mail free . . . . . . . . . . . . . . . | 48 | | I | |
| With French Military Mission to be signed " By Direction ". . . . . . . . . . . | 40 | | II | 3 |
| **Line of Communicatio ns** — | | | | |
| Advance Section : | | | | |
| Orders for transportation in, sent to regulating officer . . . . . . . . . . | 73 | | | 10 |
| Personnel engaged in transportation, subject orders regulating officer . . . | 73 | | | 12 |
| Ten day ration report submitted to C. Q. | 73 | | | 28 |
| Amended by G. O. 66 and 75 . . . . . . | | | | |
| American Red CrossMil. Hospital No. 2 under control of C. G. . . . . . . . . . . . | 51 | | | 1 |
| American Red Cross Mil. Hosp. No. 3 control of C. G. . . . . . . . . . . . . . . | 54 | | II | 1 |
| Appointed Commanding General (Amended by G. O. 75) . . . . . . . . . . . . . | 20 | | VII | |
| Arrangements for handling incoming troops. | 24 | | IV | 2 |
| Articles not on automatic supply base . . | 43 | | | 6 |
| Changes. . . . . . . . . . . . . . . . . | 43 | | | 1 |
| Commanding General . . . . . . . . . . | 66 | | I | |
| C. O. notified of assignments . . . . . | 33 | | II | 2 |

| PURPORTS | G. O. | Bul. | Sec. | Par. |
|---|---|---|---|---|
| **Line of Communications** (Cont'd) — | | | | |
| Construction work under C. O. . . . . . | 37 | | I | 1 |
| Control of troops, etc., in zone of . . . . | 26 | | I | 2 |
| Correspondence relative to shipment through Headquarters, L. O. C. . . . . | 26 | | I | 4 |
| Creation of Base Section No. 3 for handling small organizations and detachments arriving France . . . . . . . . . . . . . | 24 | | IV | 1 |
| Depots and establishments. . . . . . . . | 43 | | | 7 |
| Detraining points for units. . . . . . . . | 33 | | II | 2 |
| Displaying National Flag in Headquarters, etc. . . . . . . . . . . . . . . | 25 | | II | |
| Field Post Office (Amended by G. O. 75). | 20 | | IV | |
| Flag, National, Displaying in Headquarters, etc. . . . . . . . . . . . . . . . | 25 | | II | |
| Functions . . . . . . . . . . . . . . . . | 73 | | | 4 |
| Information concerning assignment units obtained from C. O. . . . . . . . . . | 33 | | II | 3 |
| Intercourse with French within zone of, medium of. . . . . . . . . . . . . . . | 41 | | II | 2 |
| Military Police (Amended by G. O. 75). . | 20 | | V | |
| Motor Transport Service . . . . . . . . . | 70 | | | 6 |
| New areas as sections . . . . . . . . . . | 75 | | | |
| Order, copy of, of movement of personnel, etc., over L. O. C. furnished C. G., L. O. C. . . . . . . . . . . . . . . . . | 26 | | I | 6 |
| Orders, written, furnished for duty to base or American port of entry in France . . | 26 | | I | 5 |
| Performance of functions prescribed in F. S. R. for A. C. of S. of each section of L. O. C. . . . . . . . . . . . . . . . | 26 | | I | 1 |
| Procedure under new arrangements. . . . | 24 | | IV | 3 |
| Provost Marshal Service . . . . . . . . . | 71 | | I | 4 |
| Railroad personnel, advance section . . . | 73 | | | 6 |
| Remount service, organization. . . . . . | 39 | | | 3 |
| Reporting arrival of A. E. F. at Base Port | 26 | | I | 3 |
| Report of stray or unclaimed baggage to Chief Q. M. . . . . . . . . . . . . . . | 60 | | IV | 1 |
| Report of unclaimed baggage to Chief Q. M. | 60 | | IV | 2 |
| Responsibility C. O. Advance Section . . | 73 | | | 3 |
| Responsibility C. G. as to supplies. . . . | 73 | | | 3 |
| Responsibility C. O. each base and intermediate sec. . . . . . . . . . . . . . | 73 | | | 3 |
| Responsibility transportation supplies . . | 73 | | | 1 |
| Service of military railways (Amended by G. O. 75) . . . . . . . . . . . . . . | 20 | | IV | |
| What included in this service . . . . | 20 | | IV | 1 |
| Lines of railroads considered as military railways. . . . . . . . . . . . . | 20 | | IV | 2a-c |
| Service of territorial command (Amended by G. O. 75) . . . . . . . . . . . . | 20 | | II | |
| Stock of supplies on hand . . . . . . . . | 43 | | | 10 |
| Supplies kept on hand in various depots . | 43 | | | 3 |
| Supply, sanitary and telegraph service (Amended by G. O. 75) . . . . . . . . | 20 | | III | 1 |

| PURPORTS | G.O. | Bul. | Sec. | Par. |
|---|---|---|---|---|
| **Line of Communications** (Cont'd) — | | | | |
| Base Section No. 1 | 20 | | III | 2 |
| Base Section No. 2 | 20 | | III | 3 |
| Base Section No. 3 (Amended by G.O. 66, Sec. III and G.O. 75) | 20 | | III | 4 |
| Intermediate Section | 20 | | III | 5 |
| Advance Section | 20 | | III | 6 |
| Supply depots and establishments | 43 | | | 2 |
| Territorial Limits (Amended by G. O. 75) | 20 | | I | |
| Worn and broken material to shops along the | 73 | | | 15 |
| **Lists** — | | | | |
| Alphabetical, officers, enlisted men and civilian employees | 2 | | I | |
| Officers to whom censor stamp issued | 13 | | Appx. | |
| **Mail** — | | | | |
| Addresses not located | 65 | | II | 3 |
| Addresses unknown | 65 | | II | 2 |
| Addressed to deceased officer, soldier or civilian | 44 | | III | |
| Assignment to soldiers experienced in handling | 65 | | II | 1 |
| Card index search | 65 | | II | 4 |
| Censoring | 65 | | II | 6 |
| Closing | 13 | | II | 16 |
| Deceased addressee | 63 | | II | 5 |
| Instructions for officers to examine or stamp | 13 | | II | 15 |
| Stamping, of organizations not provided with censor stamp | | | | |
| **Marine Corps** — | | | | |
| Part of Regular Army | 31 | | III | |
| Supplies furnished, accounting for | 31 | | III | 1 |
| **Marines, Payrolls of** — | | | | |
| Procedure where there is no M. C. Paymaster | 38 | | II | |
| **Medical Corps** — | | | | |
| Director Laboratories | 58 | | IV | |
| Physical examination of officers by | 67 | | IV | |
| Reserve : | | | | |
| Director, Division of General Surgery | 58 | | IV | |
| Director, Orthopedic Surgery | 58 | | IV | |
| Director, Venereal, Skin and G. U. Surgery | 58 | | IV | |
| **Medical Department** — | | | | |
| Rolls bearing other names | 55 | | III | 1 |
| **Military Railways, Service of** — | | | | |
| Lines of railroad considered as military railways | 20 | | IV | 2 |
| What included in this service | 20 | | IV | 1 |
| **Missing** — | | | | |
| Reports of | 2 | | | 3 |
| **Money Orders** — | | | | |
| Free up to fifty francs | 15 | | I | |
| Issuing and cashing by U. S. A. post office | | 1 | II | |

| PURPORTS | G. O. | Bul. | Sec. | Par. |
|---|---|---|---|---|
| **Motor cars —** | | | | |
| Marking cars of different headquarters . . | 60 | | III | 2 |
| Methoding of marking for A. E. F. . . . . | 60 | | III | 1 |
| Mixed colors, divided horizontally . . . . | 60 | | III | 3 |
| No flags to be flown from . . . . . . . . | 60 | | III | 4 |
| **Motor Transportation Service —** | | | | |
| Advance groups . . . . . . . . . . . . . . | 70 | | | 7 |
| Army M. T. S. Park. . . . . . . . . . . | 70 | | | 5 |
| Assistants. . . . . . . . . . . . . . . . | 70 | | | 2 |
| Base groups. . . . . . . . . . . . . . . | 70 | | | 9 |
| Control authorized units. . . . . . . . . | 70 | | | 4 |
| Field ambulance service depots. . . . . . | 70 | | | 12 |
| Functions. . . . . . . . . . . . . . . . | 70 | | | 1a-f |
| General. . . . . . . . . . . . . . . . . | 70 | | | 16 |
| Intermediate groups. . . . . . . . . . . | 70 | | | 8 |
| Line of Communication, organization. . . | 70 | | | 6 |
| Operation of service. . . . . . . . . . . | 70 | | | 13 |
| Organization parks . . . . . . . . . . . | 70 | | | 10 |
| Part of Q. M. C. . . . . . . . . . . . . | 70 | | | 2 |
| Requisitions. . . . . . . . . . . . . . . | 70 | | | 15 |
| Reserve stocks of spare parts, equipment, etc. . . . . . . . . . . . . . . . | 70 | | | 14 |
| Tariff rates . . . . . . . . . . . . . . . | | 20 | II | 1 |
| Training centers. . . . . . . . . . . . . | 70 | | | 11 |
| Zone of advance. . . . . . . . . . . . . | 70 | | | 3 |
| **Nurses —** | | | | |
| Allowance floor space . . . . . . . . . . | 46 | | I | 3b |
| Free postage provision. . . . . . . . . . | 54 | | III | 1 |
| Travel by rail 1st class. . . . . . . . . | 64 | | II | 1 |
| **Officers —** | | | | |
| Accountability for performance of duties.. | 32 | | | 5 |
| Accurate and complete records, statistical division for . . . . . . . . . . . . . . | 67 | | I | 1-2 |
| Acting General Staff. . . . . . . . . . . | 66<br>84 | | V<br>I | |
| Acting non-commissioned staff, C. A. C. . | 21 | | IV | |
| Aide-de-Camp . . . . . . . . . . . . . . | 21<br>1 | | IV | |
| Adjutant General and Assistant. . . . . . | 1 | | | |
| Adjutant General charged with certain duties. . . . . . . . . . . . . . . . | 67 | | I | 1 |
| Air Commander Zone of Advance, Air Service (Revoked by C. O. 80) . . . . . | 46 | | II | 3 |
| Allotments . . . . . . . . . . . . . . . | <br>53 | 5 | I<br>II | 1 |
| Allowance floor space . . . . . . . . . . | 46 | | I | 3c |
| Alphabetical list. . . . . . . . . . . . . | 2<br>25 | | <br>IV | 2 |
| Appointing necessary boards. . . . . . . | 62 | | I | 2 |
| Appointments and promotions . . . . . . | 76 | | II | 1-4 |
| Arrest of non-commissioned . . . . . . . | 29 | | III | 14-1 |
| Artillery, insignia . . . . . . . . . . . . | | 20 | II | |
| Assigned automobiles . . . . . . . . . . | 63 | | | 6 |
| Assignment staff duty . . . . . . . . . . | 1 | | | |
| Attached A. E. F. . . . . . . . . . . . . | 1 | | II | 22 |

| PURPORTS | G. O. | Bul. | Sec. | Par. |
|---|---|---|---|---|
| **Officers** (Cont'd) — | | | | |
| Automobile passes | 63 | | | 6 |
| Aviation | 1 | | | |
| Billeting | 46 | | I | 1 |
| | 67 | | I | 1 |
| Board for reporting upon suitability and fitness | 62 | | I | 1 |
| Board of : | | | | |
| Recommendations of, against severe weather conditions during service in France | 38 | | I | |
| Arctic overshoes | 38 | | I | 1 |
| Bath houses | 38 | | I | 2 |
| Blankets | 38 | | I | 3 |
| Gloves | 38 | | I | 4 |
| Horse Covers | 38 | | I | 5 |
| Latrines | 38 | | I | 6 |
| Lights | 38 | | I | 7 |
| Lumber, nails, etc. | 38 | | I | 8 |
| Rations | 38 | | I | 9 |
| Rolling kitchens | 38 | | I | 10 |
| Sales articles, additional | 38 | | I | 11 |
| Sales, commissaries | 38 | | I | 12 |
| Sentry boxes | 38 | | I | 13 |
| Shoe repairing | 38 | | I | 14 |
| Stoves | 38 | | I | 15 |
| Straw for bedding | 38 | | I | 16 |
| Trench flooring | 38 | | I | 17 |
| Trench pumps | 38 | | I | 18 |
| Card index search of mail held at headquarters | 65 | | II | 4 |
| Charge European office, War Risk Ins. duties and authority | 50 | | I | |
| Chief Air Service | 31 | | I | |
| | 66 | | II | |
| Chief Aviation : | | | | |
| Assignment for duty | 26 | | III | |
| Relieved from duty | 26 | | III | |
| Chief Engineer Officer and Assistant | 1 | | | |
| Chief of Staff and Assistants | 1 | | | |
| Chief Ordnance Officer and Assistants | 1 | | | |
| Chief Quartermaster | 21 | | III | |
| Chief Quatermaster and Assistants | 1 | | | |
| Chief Signal — to distribute time | 79 | | II | 4 |
| Chief Signal Officer and Assistants | 1 | | | |
| Chief Signal, in charge of telegraph, telephone and radio installations | 25 | | I | |
| Chief Surgeon and Assistants | 1 | | | |
| Commandant Army Aeronautical School | 60 | | II | 2 |
| Commandant Army Heavy Artillery School | 51 | | I | 3 |
| Commandant Infantry Officers' School | 41 | | I | 2 |
| Commandant Army Schools | 46 | | III | 1-0 |
| Commanders of all grades to carry out provisions of orders | 32 | | | 6 |
| Commanders of all grades to have orders read | 32 | | | 6 |

| PURPORTS | G. O. | Bul. | Sec. | Par. |
|---|---|---|---|---|
| **Officers** (Cont'd) — | | | | |
| Commanding 1st Brigade, 1st Division . . | 31 | | II | |
| C. G., Line of Communications. . . . . . | 66<br>20 | | I<br>VII | |
| Commanding 2nd Brigade, 1st Division. . | 31 | | II | |
| Commutation of Quarters . . . . . . . . | 57 | | I | 4 |
| Conduct . . . . . . . . . . . . . . . . . | 32 | | | 3 |
| Conduct on arrival in French towns or villages . . . . . . . . . . . . . . . | 59 | | II | 1 |
| Co-operation with soldiers and civilians to insure temperance and prevent ravages venereal diseases . . . . . . . . . . . | 34 | | I | 1 |
| Definition term "Commissioned Officer". | | 11 | | |
| Detailed as Acting General Staff and Members General Staff. . . . . . . . . | 58 | | III | |
| Detailed to collect and disseminate information, duties of . . . . . . . . . . . | 21 | | I | 3 |
| Determination, patriotism, loyalty and efficiency. . . . . . . . . . . . . . . . . . | 32 | | I | |
| Director Psychiatry . . . . . . . . . . . | 81 | | I | |
| Disbursing, employing upon purchases, persons paid by commission . . . . . | 53 | | IV | 1 |
| Discipline and efficiency, maximum standard of. . . . . . . . . . . . . . . . . . | 32 | | II | |
| Discussing military information . . . . . | 10 | | II | |
| Disposition of dismissed or discharged . . | 22 | | II | |
| Division Commanders, responsibility. . . | 13 | | II | 30 |
| Elimination of unfit . . . . . . . . . . . | 62 | | I | |
| For duty with G. P. B. . . . . . . . . . . | 28 | | | |
| First Corps Center of Instruction : | | | | |
| Commandant 1st Army Corps Schools . | 45 | | | 4 |
| Director Aeronautical School. . . . . . | 45 | | | 4-g |
| Director Artillery School. . . . . . . . | 45 | | | 4-b |
| Director Engineer School . . . . . . . | 45 | | | 4-c |
| Director Field Officers' School. . . . . | 45 | | | 4-h |
| Director Gas School. . . . . . . . . . . | 45 | | | 4-d |
| Director Infantry School. . . . . . . . | 45 | | | 4-a |
| Director Sanitary School. . . . . . . . | 45 | | | 4-f |
| Director Signal School. . . . . . . . . | 45 | | | 4-o |
| Free entry parcels sent to . . . . . . . . | | 1 | I | |
| General Purchasing Agent, General Purchasing Board. . . . . . . . . . . . . . | 28 | | | |
| General Staff, insignia. . . . . . . . . . | 53 | | III | |
| Identification tags. . . . . . . . . . . . | 21 | | VI | |
| Identity cards with photograph. . . . . . | 63<br>85 | | | 1<br>1 |
| Inspector General and Assistants. . . . . | 1 | | | |
| Judge Advocate and Assistants . . . . . . | 1 | | | |
| Instructions for, who examine and stamp mail | 13 | | | |
| Liaison, use of . . . . . . . . . . . . . . | 40 | | II | 8 |
| List of, to whom censor stamp will be issued. . . . . . . . . . . . . . . . . . | 13 | | | Appx. |
| Mail addressed to deceased. . . . . . . . | 44 | | III | |
| Manager of Roads, Transportation Department . . . . . . . . . . . . . . . . . | 61 | | III | |

| PURPORTS | G.O. | Bul. | Sec. | Par. |
|---|---|---|---|---|
| **Officers** (Cont'd) — | | | | |
| Medical, designated and announced. . . . | 58 | | IV | |
| Military Boards to examine and report capacity, etc. . . . . . . . . . . . . . | 62 | | I | 2 |
| Name, address and unit on outside of envelope. . . . . . . . . . . . . . . . | | 2 | I | c |
| Names not appearing in army directory. . | 44 | | IV | |
| Non-commissioned, battalion supply sergeant, machine gun battalions . . . . . | | 20 | I | |
| Not relieved of certain duties by G. O. 67 . | 67 | | I | 8 |
| Official Address. . . . . . . . . . . . . | 11 | | I | |
| Official messages . . . . . . . . . . . . | 13 | | II | 27 |
| Passports declined for wives of, ordered to France . . . . . . . . . . . . . . . . | 21 | | II | |
| Per diem allowance for commissioned . . | 76 | | III | |
| Performance of duties . . . . . . . . . . | 32 | | | 1 |
| Photographs. . . . . . . . . . . . . . . | 85 | | | 4-5 |
| Physical examination . . . . . . . . . . | 67 | | IV | |
| Provost Marshal A. E. F. . . . . . . . . . | 17 | | I | |
| Punishment for contracting venereal diseases . . . . . . . . . . . . . . . . . | 34 | | I | 7 |
| Purchasing, Red Cross. . . . . . . . . . | 28 | | | |
| Purchasing, Y. M. C. A. . . . . . . . . . | 28 | | | |
| Quartering, General Instructions. . . . . | 57 | | I | |
| Quartering, leasing suitable buildings for. | 57 | | I | 2 |
| Railway Transport. . . . . . . . . . . . | | 19 | | 29 |
| Record of all reporting or brought to A. P. M. office . . . . . . . . . . . . | 29 | | III | 4 |
| Registered at hotels or pensions, list of. . | 29 | | III | 5 |
| Registering after arrival in Paris. . . . . | 30 | | I | 3 |
| Regular Army, temporary promotion in National Army, reporting names those unable to meet requirements . . . . . . | 54 | | I | 2 |
| Reporting of, below Brigadier General . . | 29 | | III | 1 |
| Reporting on officers of a certain class . . | 62 | | I | 4 |
| Reporting on return to the U. S. . . . . . | 25 | | III | |
| Report of deaths. . . . . . . . . . . . . | 19 | | I | |
| Report on unfitness . . . . . . . . . . . | 62 | | I | 3 |
| Reserve Corps, discharge on account of age limit . . . . . . . . . . . . . . . . . | 54 | | I | 1 |
| Saluting. . . . . . . . . . . . . . . . . | 23 | | I | 5 |
| Shelter . . . . . . . . . . . . . . . . . | 57 | | I | 1 |
| Shipment field allowance by "Grande Vitesse" . . . . . . . . . . . . . . . . . | 65 | | III | |
| Sign names on envelopes without rank . . | | 2 | I | a |
| Sign names in wrong place on envelopes. | | 2 | I | b |
| Student, Army Gen. Staff College, Billeting . . . . . . . . . . . . . . . . . . | 46 | | III | 6 |
| Suitability and fitness . . . . . . . . . . | 62 | | I | 1 |
| Supervision of, in cities . . . . . . . . . | 29 | | III | |
| Supply . . . . . . . . . . . . . . . . . | | 19 | | 1 |
| Temporary Disbursing, Transfer of Balances . . . . . . . . . . . . . . . . . | 19 | | II | |
| Tentage, allowance . . . . . . . . . . . | 57 | | I | 3 |
| To be advised of instructions for reporting. | 25 | | III | 1 |

| PURPORTS | G.O. | Bul. | Sec. | Par. |
|---|---|---|---|---|
| **Officers** (Cont'd) — | | | | |
| Training of troops | 32 | | | 4 |
| Travel by rail : | | | | |
| Commissioned, 1st class | 64 | | II | 1 |
| Non-commissioned, 2nd class | 64 | | II | 1 |
| Visiting vicinity French and British front. | 19 | | III | |
| Visiting French and British army zones | 63 | 4 | | |
| Violation G.O. 13 | | 2 | I | |
| Written permission to visit Paris | 30 | | I | 2 |
| **Officers' Reserve Corps** — | | | | |
| Discharging officers on account of age limit | 54 | | I | 1 |
| Discharging soldiers receiving commissions in | 68 | | V | 2 |
| **Oil, Fuel** — | | | | |
| Economy in use | 40 | | I | 1 |
| Intermediary between A.E.F. and French Government, concerning supply | 40 | | I | 3 |
| Requisitions | 40 | | I | 4 |
| **Oil, Lubricants** — | | | | |
| Economy in use | 40 | | I | 1 |
| Intermediary between A.E.F. and French Government, concerning supply | 40 | | I | 3 |
| Requisitions | 40 | | I | 4 |
| **Operation** — | | | | |
| Remount Service | 39 | | | 8 |
| **Orders, General** — | | | | |
| (See **General Orders** —) | | | | |
| **Organization, A.E.F.** | 1 | | | |
| **Organization** — | | | | |
| Remount Service | 39 | | | |
| Tables of (See **Tables of Organization**) — | | | | |
| **Passes** — | | | | |
| American citizens | 29 | | III | 24 |
| Automobile | 63 | | | 8 |
| Automobile, blue | 63 | | | 6 |
| List of, required | 63 | | | 8 |
| Return of cancelled | 63 | | | 6 |
| Special pink, for chauffeurs of War Dept. autos | 63 | | | 6 |
| Transient troops | 77 | | | 6 |
| **Passports** — | | | | |
| Declined for wives of officers ordered to France | 21 | | II | |
| **Payrolls of Marines** — | | | | |
| Procedure where there is no M.C. Paymaster | 38 | | | 2 |
| **Per Diem Allowances** — | | | | |
| Civilian employees in France | 74 | | III | 3 |
| Commissioned officers and enlisted personnel | 76 | | | 3 |
| Of $ 2.00 to civilian employees and field clerks | 35 | | II | 1 |
| **Personnel — Remount Service** — | | | | |
| Advance Remount Depots | 39 | | | 4a |

| PURPORTS | G.O. | Bul. | Sec. | Par. |
|---|---|---|---|---|
| **Personnel — Remount Service** (Cont'd) — | | | | |
| Advance Veterinary Hospitals . . . . . . | 39 | | | 4b |
| Base Remount Depots . . . . . . . . . . | 39 | | | 6a |
| Base Veterinary Hospitals . . . . . . . . | 39 | | | 6b |
| **Physical Examination** — | | | | |
| Conducted by two officers, Medical Corps. | 67 | | IV | |
| Officers. . . . . . . . . . . . . . . . | 67 | | IV | |
| **Police, Military** — | | | | |
| (See **Military Police** —) | | | | |
| **Police Regulations** — | | | | |
| Local, conforming to by Military Police . | 29 | | I | |
| **Postage** — | | | | |
| Forwarding correspondence without prepayment of . . . . . . . . . . . . . | 13 | | II | 13-1 |
| Free provision (Amended by G.O. 68) . . | 54 | | III | 2 |
| Free provision not applying letters certain civilians. . . . . . . . . . . . . . . | 68 | | I | 1 |
| Not required in France by A.E.F. . . . . | 15 | | I | |
| Stamps, where sold and use . . . . . . . | | 1 | II | |
| **Post Offices** — | | | | |
| Civil, use of. . . . . . . . . . . . . . | 13 | | II | 18 |
| Field . . . . . . . . . . . . . . . . . | 20 | | VI | |
| Money Orders. . . . . . . . . . . . . . | | 1 | II | |
| Postage stamps, selling and use . . . . . | | 1 | II | |
| **Promotions and Appointments** — | | | | |
| Consideration Officer's record . . . . . . | 76 | | II | 3 |
| Recommendations . . . . . . . . . . . . | 76 | | II | 1 |
| Special Regulations . . . . . . . . . . . | 76 | | II | 4 |
| Temporary . . . . . . . . . . . . . . . | 76 | | II | 2 |
| Temporary, officers Regular Army in National Army, reporting names those unable to meet requirements. . . . . . | 54 | | I | 1 |
| **Property** — | | | | |
| Accounting for . . . . . . . . . . . . . | 74 | | I | 6 |
| Disinfecting, cleaning and repairing . . . | 68 | | IV | 4 |
| Final returns . . . . . . . . . . . . . . | 74 | | I | 1 |
| Invoices and Receipts . . . . . . . . . . | 68 | | IV | 2 |
| | 74 | | I | 4 |
| Misuse, abuse and waste. . . . . . . . . | 74 | | II | |
| No unserviceable to be destroyed. . . . . | 68 | | IV | 3 |
| Number copies final return and disposition. | 74 | | I | 3 |
| Physical inventory balance on hand . . . | 74 | | I | 2 |
| Private, damage to . . . . . . . . . . . | 7 | | | |
| Turning in unserviceable to Quartermaster. | 68 | | IV | 1 |
| Unserviceable Q.M. sent to Q.M. Depots. | 68 | | IV | 3 |
| **Prophylactic Stations** — | | | | |
| (See **Diseases, Venereal**) — | | | | |
| **Psychiatry** — | | | | |
| Director. . . . . . . . . . . . . . . . | 81 | | I | |
| **Purchasing Board, General** — | | | | |
| Bringing order to attention officers making requisitions or purchases. . . . . . . . | 66 | | IV | 6 |
| Employing upon purchases persons paid by commission . . . . . . . . . . . . . . | 53 | | IV | |
| Orders to purchasing agents. . . . . . . | 66 | | IV | |

| PURPORTS | G. O. | Bul | Sec. | Par. |
|---|---|---|---|---|
| **Purchasing Board, General** (Cont'd) — | | | | |
| Purchases subject approval. . . . . . . . . | 66 | | IV | 2 |
| Requisitions for material upon French Government made through. . . . . . . | 66 | | IV | 1 |
| Rules for handling small purchases at Paris and General Headquarters . . . . | 66 | | IV | 3 |
| Special branch to handle emergency and telegraphic requisitions . . . . . . . . | 66 | | IV | 4 |
| System of co-ordination of American purchases. . . . . . . . . . . . . . . . . | 53 | | IV | |
| **Quartering Officers** — | | | | |
| General instructions. . . . . . . . . . . | 57 | | I | |
| **Quarters** — | | | | |
| Commutation of officers . . . . . . . . . . | 57 | | I | 4 |
| Officers : Leasing of suitable buildings for. | 57 | | I | 2 |
| **Railroads** — | | | | |
| French Military rate for travel thereon. . | 33 | | I | |
| Identity cards give right to railway warrant or ticket . . . . . . . . . . . . . | 63 | | | 7 |
| **Railway Tickets** — | | | | |
| Stamp of A. P. M. on papers necessary for purchase . . . . . . . . . . . . . . . | 29 | | III | 3 |
| **Railway Transportation Officers** . . . . . . | | 19 | | |
| **Rations** — | | | | |
| Articles and quantities issued . . . . . . | | 19 | | 27 |
| Barter or sale prohibited. . . . . . . . . | | 18 | I | 7 |
| Commutation of, for patients of hospitals, voucher for . . . . . . . . . . . . . . | 24 | | I | 1 |
| Commutation of, when paid at rate of $2.00 per day : | | | | |
| Enlisted chauffeurs. . . . . . . . . . . | 42 | | II | 1 |
| Soldiers. . . . . . . . . . . . . . . . . | 42 | | II | 2 |
| Daily issue of. . . . . . . . . . . . . . | 18 | | II | 6 |
| Field . . . . . . . . . . . . . . . . . . . | 18 | | II | 2 |
| Field and reserve, supplemented by garrison ration. . . . . . . . . . . . . . . | 18 | | II | 1-1 |
| Flour component of garrison. . . . . . . | 78 | | IV | |
| Fresh beef, issue of . . . . . . . . . . . | 18 | | IV | 2 |
| Fresh vegetables, issue of . . . . . . . . | 18 | | II | 5 |
| Garrison . . . . . . . . . . . . . . . . . | 18 | | II | 1 |
| Interpreters entitled. . . . . . . . . . . | 41 | | IV | 5 |
| Issue figs and dates as substitutive articles (Amended by G. O. 78) . . . . . . . . . | 67 | | III | |
| Issue raisins as substitutive article. . . . | 78 | | III | |
| Reserve. . . . . . . . . . . . . . . . . . | 18 | | II | 3 |
| | 38 | | 1 | 9 |
| Substitution in garrison . . . . . . . . . . | 26 | | IV | |
| **Red Cross** — | | | | |
| Employing upon purchases, persons paid by commission . . . . . . . . . . . . . | 53 | | IV | 1 |
| **Red Cross, American** — | | | | |
| Bureau of information of casualties : | | | | |
| Information obtained by searchers . . . | 81 | | II | 1 |
| Reports of searchers. . . . . . . . . . | 81 | | II | 2 |
| Work by mail instead cable . . . . . . | 81 | | II | 3 |

| PURPORTS | G. O. | Bul. | Sec. | Par. |
|---|---|---|---|---|
| **Red ross, American** — (Cont'd) | | | | |
| Members allowed medical and hospital attention and privileges purchasing medicines. . . . . . . . . . . . . . . . . | 61 | | II | |
| Visiting French or British army zones . . | 63 | | IV | |
| " Workers' Permits ", General instructions. | 63 | | | 3 |
| **Red Cross, American and Y. M. C. A.** — | | | | |
| Activities of, in U. S. A. hospitals. . . . . | 48 | | II | |
| **Red Cross and Y. M. C. A** — | | | | |
| Activities of, in U. S. A. hospitals. . . . . | 48 | | II | |
| Chaplains calling upon one agency to perform functions of another . . . . . . . | 26 | | II | 5 |
| Commanding Officers to bring needs to attention of . . . . . . . . . . . . . . | 26 | | II | 3 |
| Co-operation of chaplains with . . . . . | 26 | | II | 3 |
| Details regarding equipment and personnel to be settled by conference between senior representatives . . . . . . | 26 | | II | 2 |
| Former to provide for relief work . . . . | 26 | | II | 1 |
| Furnishing of assistance by one to other. | 26 | | II | 4 |
| G. O. 26, Sec. 2 amplified (G. O. 48, Sec. I). | | | | |
| Latter to provide for amusement and recreation . . . . . . . . . . . . . . . . . | 26 | | II | 1 |
| Partition activities not intended to give monopoly to either organization, but as a guiding rule. . . . . . . . . . . . . . | 26 | | II | 1 |
| Purchasing and Disbursing Officer. . . . | 28 | | | |
| **Registering of Officers and soldiers after arrival in Paris** . . . . . . . . . . . | 30 | | I | 3 |
| **Remount Service** — | | | | |
| Accountability. . . . . . . . . . . . . . | 39 | | | 8-a |
| Advance groups . . . . . . . . . . . . . | 39 | | | 4 |
| Advance Remount Depots . . . . . . . . | 39 | | | 4-a |
| Advance Veterinary, Hospitals, Personnel. | 39 | | | 4-b |
| Attached to Q. M. C. . . . . . . . . . . | 39 | | | 1 |
| Base Groups . . . . . . . . . . . . . . | 39 | | | 6 |
| Base Remount Depots, personnel. . . . . | 39 | | | 6-a |
| Base Veterinary Hospitals, personnel. . . | 39 | | | 6-b |
| Duties . . . . . . . . . . . . . . . . . | 39 | | | 1 |
| Horseshoes and farriers . . . . . . . . . | 39 | | | 7-a |
| Intermediate Group . . . . . . . . . . . | 39 | | | 5 |
| Intermediate Remount Depots . . . . . . | 39 | | | 5-a |
| Intermediate Veterinary Hospitals . . . . | 39 | | | 5-b |
| Line of Communication, organization. . . | 39 | | | 3 |
| Operation. . . . . . . . . . . . . . . . | 39 | | | 8 |
| Organization . . . . . . . . . . . . . . | 39 | | | |
| Other necessary. . . . . . . . . . . . . | 39 | | | 7-b |
| Remount Depots. . . . . . . . . . . . . | 39 | | | 8-a |
| Schools. . . . . . . . . . . . . . . . . | 39 | | | 7 |
| Stray animals. . . . . . . . . . . . . . | 39 | | | 8-a |
| Veterinary hospitals. . . . . . . . . . . | 39 | | | 8-b |
| Veterinary hospitals, mobile and stationary, attached to . . . . . . . . . . . . | 39 | | | 1 |
| Zone of Advance. . . . . . . . . . . . . | 39 | | | 2 |

| PURPORTS | G. O. | Bul. | Sec. | Par. |
|---|---|---|---|---|
| **Reports —** | | | | |
| Death of officer | 19 | | | 1 |
| Deaths, discharges or desertion of enlisted men | 19 | | | 1 |
| Enumerating those to be submitted | 24 | | II | 1-6 |
| Forwarding copies of, required by Par. 916 and 918, A. R | 19 | | IV | |
| Killed, wounded and missing | 2 | | | 3 |
| Military operations | 21 | | I | 2 |
| On supplies | | 19 | | |
| (See **Operation Orders**) — | | | | |
| Theft and damage claims | 29 | | III | 25 |
| Unclaimed baggage | 60 | | IV | 2 |
| **Requisitions —** | | | | |
| Fuel wood, coal, gasoline, lubricating oils, fuel oil and greases | 40 | | I | 1 |
| Funds required to purchase text books | | 6 | I | 2 |
| Supplies for Motor Transport Service | 70 | | | 15 |
| **Requisitions —** | | | | |
| Bringing order to attention officers making requisitions or purchases | 66 | | IV | 6 |
| For spare parts, etc., for M. T. Service | 70 | | | 15 |
| Handling emergency and telegraphic | 66 | | IV | 4 |
| Orders to Purchasing Agent | 66 | | IV | 5 |
| Purchases subject to approval Genl. Purch. Board | 66 | | IV | 2 |
| Rules for handling small purchases at Paris and GHQ | 66 | | IV | 3 |
| Upon French Gov't for material thru G. P. B. | 66 | | IV | 1 |
| **Rooms —** | | | | |
| May be leased for cooking or messing when officers are billeted or quartered in dormitories | 57 | | I | 3 |
| **Salvation Army —** | | | | |
| Members allowed medical and hospital attendance and privilege of purchasing medicines | 61 | | II | |
| Traveling in automobiles | 63 | | | 6 |
| Visiting French and British army zones | 63 | | | 4 |
| "Worker's Permit", general instructions | 63 | | | 3 |
| **Sam Browne Belt —** | 23 | | II | |
| **Sanitary, Supply and Telegraph Service, L. O. C. —** | | | | |
| Advance Section | 20 | | III | 6 |
| Base Section N° 1 | 20 | | III | 2 |
| Base Section N° 2 | 20 | | III | 3 |
| Base Section N° 3 | 20 | | III | 4 |
| Intermediate Section | 20 | | III | 5 |
| **Sanitary Service —** | | | | |
| Free postage provision for those forming part of | 54 | | III | 1 |
| **Sick and Wounded —** | | | | |
| Instruction for the disposition of (Amended by G. O. 71) | 44 | | V | 1-4 |

| PURPORTS | G. O. | Bul. | Sec. | Par. |
|---|---|---|---|---|
| **Signatures** — | | | | |
| Counter, for officer and censor stamp on official envelopes | 22 | | III | |
| **Sites** — | | | | |
| For burial grounds | 27 | | | 4-k |
| **Special Hospitals for Venereal Cases** | 34 | | I | 6 |
| **Spurs** — | | | | |
| Wearing of | 23 | | I | 2-2 |
| **Spy System** | | | | |
| Enemy, for gathering and forwarding military information | 10 | | | 1 |
| **Statistics** — | | | | |
| Correspondence between various elements statistical services | 67 | | I | 6 |
| Duties of statistical sections | 67 | | I | 4-a-g |
| Officers, soldiers, civilians and prisoners of war | 67 | | I | 1 |
| Preparation detailed instructions, blank forms, etc | 67 | | I | 7 |
| Statistical division, established and duties | 67 | | I | 2-a-e |
| Statistical sections, where established | 67 | | I | 3-a-m |
| Statistical sub-sections, organization and personnel | 67 | | I | 5-a-i |
| **Supplies** — | | | | |
| Accounting for | 43 | | | 10 |
| Agencies | 73 | | | 6 |
| Anti-Gas | 53 | | I | 1 |
| Articles not on automatic supply basis | 43 | | | 6 |
| Cars : | | | | |
| Arriving at destination unsealed | | 19 | | 18 |
| Deficiency in contents | | 19 | | 19 |
| Discrepancies in number packages, etc. | | 19 | | 22 |
| Economizing loading capacity | | 19 | | 4 |
| Labeling | | 19 | | 8 |
| Non-arrival | | 19 | | 23 |
| Number and kind for forwarding | | 19 | | 10 |
| Numbers, contents and destination list. | | 19 | | 11 |
| Receipt and unloading | | 19 | | 15 |
| Receipt for received | | 19 | | 21 |
| Record number received, date of receipt, etc | | 19 | | 28 |
| Record of all billed | | 19 | | 34 |
| Removing labels, etc | | 19 | | 20 |
| Reporting delay, receipt for shipment. | | 19 | | 14 |
| Report of, on hand, received, made empty, etc | | 19 | | 30 |
| Report of, on hand unreleased | | 19 | | 31 |
| Reports of number received and forwarded each day | | 19 | | 33 |
| Sealing | | 19 | | 6 |
| Subsequent arrival detached | | 19 | | 24 |
| Unloading | | 19 | | 16 |
| Cases or sacks; broken packages | | 19 | | 26 |
| Checkers list, placing | | 19 | | 3 |
| Chiefs of Supply Dept., responsibility | 73 | | | 2 |

| PURPORTS | G. O. | Bul. | Sec. | Par. |
|---|---|---|---|---|
| **Supplies** (Cont'd) — | | | | |
| Classification and distribution of supplies. | 73 | | | 16 |
| Consignor, liability . . . . . . . . . . . | | 19 | | 13 |
| Class 1 . . . . . . . . . . . . . . . . . | 73 | | | 17-a-f |
| Class 2, shipments of less than carload lots. . . . . . . . . . . . . . . . . | | 19 | | 5 |
| Class 2, (shoes, clothing, etc.) . . . . . . | 73 | | | 20-a-c |
| Class 3, (wagons, trucks, axes, shovels, etc.) . . . . . . . . . . . . . . . . . | 73 | | | 22 |
| Class 4, ammunition, timber, etc. . . . . | 73 | | | 24 |
| Delivery of, in advance of requisitions . . | 43 | | | 4 |
| Depots and establishments, functions of chief in charge. . . . . . . . . . . . . | 43 | | | 7 |
| Detraining, re-embarking and forwarding troops. . . . . . . . . . . . . . . . . | 73 | | | 9 |
| Director General of Transportation. . . . | 73 | | | 5 |
| Divided into three phases; responsibility for each. . . . . . . . . . . . . . . . | 73 | | | 1 |
| Double checking. . . . . . . . . . . . . | | 19 | | 2 |
| Evacuation station. . . . . . . . . . . . | 73 | | | 14 |
| Expediting Class 4 supplies . . . . . . . | 73 | | | 25 |
| Freight, responsibility for delivery . . . . | | 19 | | 12 |
| Full stock to be kept on hand by C. G., L. O. C. . . . . . . . . . . . . . . . . | 43 | | | 3 |
| Furnished Marine Corps . . . . . . . . . | 31 | | III | 1 |
| Gasoline and lubricating oil . . . . . . . | 70 | | | 5 |
| Issued from depots to troops. . . . . . . | 43 | | | 10 |
| Line of Communications : | | | | |
| Advance Section : | | | | |
| Orders for transportation in, sent to Regulating officer . . . . . . . . . | 73 | | | 10 |
| Personnel engaged in transportation subject orders Regulating officer . . | 73 | | | 12 |
| Ten day ration reports submitted to C. O. . . . . . . . . . . . . . . | 73 | | | 28 |
| Functions . . . . . . . . . . . . . . . . | 73 | | | 4 |
| Railroad personnel, Advance Section . . | 73 | | | 6 |
| Responsibility C. O., Advance Section . | 73 | | | 3 |
| Responsibility C. G. . . . . . . . . . | 73 | | | 3 |
| Responsibility C. O. each Base and Intermediate Section. . . . . . . . . | 73 | | | 3 |
| Responsible transportation supply . . . | 73 | | | 1 |
| Worn out and broken material to shops along the . . . . . . . . . . . . . | 73 | | | 15 |
| Lists of special or unusual. . . . . . . . | 43 | | | 4 |
| Local Supply Officer. . . . . . . . . . . | 73 | | | 27 |
| Local Supply Officer, duties . . . . . . . | | 19 | | 17 |
| Marking bulk train shipments . . . . . . | | 19 | | 9 |
| Of American Troops in France. . . . . . | 73 | | | 1 |
| Old pasters, disposition . . . . . . . . . | | 19 | | 7 |
| Ordre de Transport . . . . . . . . . . . | | 19 | | 35 |
| Organizations and individuals demanding excess amounts . . . . . . . . . . . . | 43 | | | 10 |
| Over Issues and under Issues. . . . . . . | 73 | | | 19 |
| Perishable, issue. . . . . . . . . . . . . | | 19 | | 25 |

| PURPORTS | G. G. | Bul. | Sec. | Par. |
|---|---|---|---|---|
| **Supplies** (Cont'd) — | | | | |
| Precautions to insure safe arrival in sound condition . . . . . . . . . . . . . . . | | 19 | | 1 |
| Procedure in completing requisitions . . . | 73 | | | 23 |
| Procedure on arrival of bulk trains. . . . | 73 | | | 18 |
| Prompt receipt . . . . . . . . . . . . . | 43 | | | 1 |
| Purchase by P. O., G. P. B. . . . . . . | 43 | | | 8 |
| Railheads and refilling points . . . . . | 73 | | | 15 |
| Railroad personnel. . . . . . . . . . . | 73 | | | 6 |
| Railway Transport Officers. . . . . . . . | | 19 | | 29 |
| Regulating Officers : | | | | |
| Close touch with headquarters, army, communications . . . . . . . . . . . | 73 | | | 11 |
| Duties. . . . . . . . . . . . . . . . . | 73 | | | 10 |
| Personnel engaged in transportation subject to orders of. . . . . . . . . | 73 | | | 12 |
| Regulating Station. . . . . . . . . . . . | 73 | | | 8 |
| Replacement of . . . . . . . . . . . . . | 43 | | | 6 |
| Requisition of corps troops and army troops. . . . . . . . . . . . . . . . . | 73 | | | 26 |
| Responsibility for accuracy, etc., of requisitions . . . . . . . . . . . . . . . . . | 73 | | | 21 |
| Responsibility for procurement. . . . . . | 43 | | | 3 |
| Shipment of. . . . . . . . . . . . . . . | | 19 | | |
| Shortage . . . . . . . . . . . . . . . . | 43 | | | 5 |
| Sorting Stations . . . . . . . . . . . . . | 73 | | | 15 |
| Supply Depots and establishments, command of. . . . . . . . . . . . . . . . | 43 | | | 2 |
| Supply Depots, duties of officer in charge. | 43 | | | 9 |
| Supply Officers, local and divisional, responsibility. . . . . . . . . . . . . . . | | 19 | | 27 |
| Technical inspections . . . . . . . . . . | 43 | | | 11 |
| To be obtained from Europe . . . . . . . | 43 | | | 3 |
| To troops to be expedited . . . . . . . . | 73 | | | 25 |
| Troop trains, reports of arrival or departure. | | 19 | | 32 |
| Waste and misuse of. . . . . . . . . . . | 43 | | | 10 |
| Woolen spiral puttees, issue . . . . . . . | | 2 | II | |
| **Supply** — | | | | |
| Sanitary and telegraph service, L. O. C. (Amended by G. O. 75) . . . . . . . . | 20 | | | |
| Advance Section. . . . . . . . . . . | 20 | | III | 6 |
| Base Section No. 1. . . . . . . . . . | 20 | | III | 2 |
| Base Section No. 2. . . . . . . . . . | 20 | | III | 3 |
| Base Section No. 3. . . . . . . . . . | 20 | | III | 4 |
| Intermediate Section. . . . . . . . . | 20 | | III | 5 |
| **Supply Depots** — | | | | |
| L. O. C., control and supervision of . . . | 43 | | | 3 |
| L. O. C., establishment and personnel . . | 43 | | | 2 |
| L. O. C., inspection of. . . . . . . . . . | 43 | | | 11 |
| **Supply Officers** — | | | | |
| Responsibility of. . . . . . . . . . . . . | | 19 | | |
| Will submit ration returns to division Q. M. or Q. M. of organization to which they are attached . . . . . . . . . . . | 73 | | | 17-a |
| **Supply Sanitary and Telegraph Service** — . | 20 | | III | |

| PURPORTS | G. O. | Bul. | Sec. | Par. |
|---|---|---|---|---|
| **Surgeons, Veterinary —** | | | | |
| (See **Veterinary Surgeons**) — | | | | |
| **Syphilitic Register to be Kept —** . . . . . . | 34 | | I | 4 |
| **Telegrams —** | | | | |
| From United States . . . . . . . . . . . . | 13 | | II | 26 |
| **Telegraph —** | | | | |
| Civil . . . . . . . . . . . . . . . . . . . . | 13 | | II | 24 |
| Military . . . . . . . . . . . . . . . . . . | 25 | | V | |
| **Telegraph, Telephone, Radio, etc. —** | | | | |
| Under supervision of Chief Signal Officer. | 25 | | I | |
| **Telegraph Installations —** | | | | |
| Chief Signal Officer in Charge. . . . . . | 25 | | I | |
| **Telegraph, Sanitary and Supply Service, L. O. C. —** | | | | |
| Advance Section. . . . . . . . . . . . . . | 20 | | III | 6 |
| Base Section No. 1. . . . . . . . . . . . . | 20 | | III | 2 |
| Base Section No. 2. . . . . . . . . . . . . | 20 | | III | 3 |
| Base Section No. 3 (Amended by G. O. 66. Sec. III, Par. 1 and G. O. 75). . . . . | 20 | | III | 4 |
| Intermediate Section. . . . . . . . . . . . | 20 | | III | 5 |
| **Telegraphic Censorship**. . . . . . . . . . . | 13 | | | |
| **Telegraphic Lines —** | | | | |
| Censorship . . . . . . . . . . . . . . . . . | 13 | | II | 22 |
| Franking privilege over French. . . . . . | 21 | | V | |
| **Telephone Communications**. . . . . . . . . | 37 | | II | |
| **Telephone Installations —** | | | | |
| Chief Signal officer in Charge . . . . . | 25 | | I | |
| **Telephone Lines —** | | | | |
| Authorization and arrangements for use of English in communications over French lines . . . . . . . . . . . . . . . . . . | 37 | | II | |
| **Telephonic Censorship** . . . . . . . . . . . | 13 | | II | 28 |
| **Temperance —** | | | | |
| Co-operation of officers, soldiers and civilians to insure . . . . . . . . . . . . . . | 34 | | I | 1 |
| **Temporary Appointments and Promotions Pertaining to 1st Corps**. . . . . . . . . . | 64 | | III | |
| **Temporary Disbursing Officer**. . . . . . . . | 19 | | | |
| **Tentage for Officers —** | | | | |
| Allowance. . . . . . . . . . . . . . . . . . | 57 | | I | 3 |
| **Terms —** | | | | |
| " Commissioned Officer ", definition. . . | | 11 | | |
| In War Risk Ins. Act, definition . . . . . | | 12 | | |
| **Theft and Damage Claims —** | | | | |
| Report of . . . . . . . . . . . . . . . . . | 29 | | III | 25 |
| **Treasury Balances —** | 19 | | | |
| **Treatment —** | | | | |
| Of men having venereal diseases. . . . . | 77 | | | 5 |
| **Troops —** | | | | |
| Allowance floor space . . . . . . . . . . . | 46 | | I | 3 |
| Enlisted men . . . . . . . . . . . . . . . | 46 | | I | 3a |
| Hospitals . . . . . . . . . . . . . . . . . | 46 | | I | 3d |
| Nurses . . . . . . . . . . . . . . . . . . . | 46 | | I | 3b |
| Officers. . . . . . . . . . . . . . . . . . . | 46 | | I | 3c |
| American, maintenance of . . . . . . . . | | 10 | | |

| PURPORTS | G. O. | Bul. | Sec. | Par. |
|---|---|---|---|---|
| **Troops** (Con'd) — | | | | |
| And members of crew not permitted ashore until report is submitted. . . . . . . . | 77 | | | 5 |
| Arrangements for handling incoming. . . | 24 | | IV | 2 |
| Arrival at station — arrangement for. . . | 37 | | II | |
| Arrival of, notification from Military Attaché in London. . . . . . . . . . . | 24 | | IV | 3b |
| Arrival on vessels or transports — examination of . . . . . . . . . . . . . . . | 77 | | | 5 |
| Billeting officers, men and other persons . | 46 | | I | 1 |
| Billet arrangement. . . . . . . . . . . . | 46 | | I | 2 |
| Construction for sheltering. . . . . . . . | 46 | | I | 1 |
| Control of, in zone of Line of Communication. . . . . . . . . . . . . . . . . | 26 | | I | 2 |
| Correction of irregularities and deficiencies at inspections. . . . . . . . . . . . . | 57 | | III | 1 |
| Divisional, corps and army assigned to 1st corps . . . . . . . . . . . . . . . | 64 | | III | 1 |
| Information as to organization of command and territorial services in areas occupied by Americans . . . . . . . . . . . . . | | 10 | | |
| Lower bunk frames, clearance . . . . . . | 46 | | I | 4 |
| Maintenance, American . . . . . . . . . | | 10 | | |
| Passes for transient . . . . . . . . . . . | 77 | | | 6 |
| Puttees, woolen spiral, issue of. . . . . . | | 2 | II | |
| Recommendations involving certain changes, forwarding of. . . . . . . . . . . | 57 | | III | 3 |
| Statement of certain irregularities and deficiencies at inspections, forwarding of. . . . . . . . . . . . . . . . . . . . | 57 | | III | 2 |
| Supplies for. . . . . . . . . . . . . . . | 73 | | | |
| Storehouse, erection. . . . . . . . . . . | 46 | | I | 1 |
| Stove allowance amended . . . . . . . . | 46 | | I | 5 |
| Training of . . . . . . . . . . . . . . . | 32 | | | 4 |
| **Typhoid Prophylaxis** — | | | | |
| Inspection of service record for. . . . . . | 84 | | II | |
| **Unserviceable Q. M. property will be sent to Q. M. Depots** . . . . . . . . . . . . | 68 | | IV | 3 |
| **Venereal Diseases** — | | | | |
| Advice and warning against . . . . . . . | 34 | | I | 3 |
| Base Surgeon will co-operate with urologist for the prevention of. . . . . . . . | 77 | | | 4 |
| Daily report required of new cases. . . . | 77 | | | 9 |
| Medical officers to co-operate with municipal. . . . . . . . . . . . . . . . . . . | 34 | | I | 8 |
| Names of women to be ascertained . . . . | 34 | | I | 8 |
| Necessity of early treatment to be impressed upon men. . . . . . . . . . . . . . . | 77 | | | 7 |
| Officers contracting venereal diseases. . . | 34 | | I | 7 |
| See **Diseases, venereal** — | | | | |
| **Veterinary Hospitals** — | | | | |
| Remount Service : | | | | |
| Advance, personnel . . . . . . . . . . | 39 | | | 4b |
| Base, personnel. . . . . . . . . . . . . | 39 | | | 6b |
| Hospital accommodations . . . . . . . | 39 | | | 8b |

| PURPORTS | G. O. | Bul. | Sec. | Par. |
|---|---|---|---|---|
| **Veterinary Hospitals** (Cont'd) — | | | | |
| Intermediate . . . . . . . . . . . . . | 39 | | | 5b |
| Mobile and stationary . . . . . . . . . | 39 | | | 1 |
| Purpose . . . . . . . . . . . . . . . . | 39 | | | 8b |
| **Veterinary Surgeons** — | | | | |
| Animals undergoing treatment . . . . . . | 42 | | I | 2 |
| Assistants to . . . . . . . . . . . . . | 42 | | I | 1 |
| **Vouchers for Commutation of Rations** — | | | | |
| For patients of hospitals . . . . . . . . | 24 | | | 1 |
| **Wages paid French Labor** — | 48 | | III | 2 |
| **War Risk Insurance** — | | | | |
| Allotments, compulsory and voluntary : | | | | |
| Enlistments prior to Nov. 1st. . . . . . | | 17 | | |
| Pro-rating . . . . . . . . . . . . . . . | | 17 | | |
| Application blanks . . . . . . . . . . . | 56 | | | 2 |
| Application, third parties for . . . . . . | | 16 | | |
| Applications, inspection . . . . . . . . . | | 12 | | |
| Benefits in addition to . . . . . . . . . | | 12 | | |
| Bill, compulsory allotment . . . . . . . . | | 5 | I | |
| Changes in instructions . . . . . . . . . | 61 | | | 4 |
| Clerks, field, army, and Q. M. C.. entitled to benefits . . . . . . . . . . . . . . | | 14 | II | |
| Completed applications : | | | | |
| Inspection . . . . . . . . . . . . . . . | 56 | | | 6 |
| Where to be mailed . . . . . . . . . . | 56 | | | 6 |
| Where to be sent . . . . . . . . . . . | 56 | | | 6 |
| Date application, amount and premium. . | 56 | | | 4 |
| Dead, discharged and deserted soldiers. . | | 16 | | 2 |
| Dependents, names and addresses . . . . | 56 | | | 3 |
| Emergency application blanks . . . . . . | 56 | | | |
| Establishment of branch offices . . . . . . | 50 | | I | |
| Extracts from War Department . . . . . . | 56 | | | |
| Granting . . . . . . . . . . . . . . . . | 56 | | I | |
| Officer in charge of European office, duties and authority . . . . . . . . . . . . . | 50 | | | 1 |
| Paying premiums . . . . . . . . . . . . | 56 | | | 7 |
| Premiums, full monthly, withholding . . | | 17 | | |
| Premiums withheld, abstract . . . . . . . | | 18 | | |
| Premiums withheld from pay . . . . . . | 65 | | | 5 |
| Rates, premium . . . . . . . . . . . . . | | 11<br>12 | | |
| Regular and Liberty Loan allotments, abstract . . . . . . . . . . . . . . . . | | 14 | | |
| Regulations to carry out provisions of | 56 | | | |
| Reporting funds received from premiums . | 56 | | | 8 |
| Table and explanatory notes . . . . . . . | | 11 | | |
| Terms in Act, definition . . . . . . . . . | | 12 | | |
| Total disability benefits . . . . . . . . . | | 14 | | 1 |
| When increase in premiums take place . . | | 14 | | 1 |
| “ **Worker's permit** ” — | | | | |
| General Instructions . . . . . . . . . . . | 63<br>85 | | | 3<br>1 |
| Application for . . . . . . . . . . . . . | 63 | | | 3 |
| Shall be issued and signed by A. G., A. E. F | 63 | | | 3 |
| Shall have photo attached . . . . . . . . | 63 | | | 3 |

| PURPORTS | G. O. | Bul. | Sec. | Par. |
|---|---|---|---|---|
| **" Worker's Permit " —** | | | | |
| Will be withdrawn from members of Red Cross, Y. M. C. A., etc., when relieved from duty . . . . . . . . . | 63 | | | 3 |
| **Wounded —** | | | | |
| Disposition of. . . . . . . . . . . . . . | 44 | | | |
| Reports of. . . . . . . . . . . . . . . . | 2 | | | 3 |
| **Wounded and Sick —** | | | | |
| Instructions as to disposition (Amended by G. O. 71) . . . . . . . . . . . . . | 44 | | V | 1-4 |
| **Y. M. C. A. and Red Cross —** | | | | |
| Activities of, in U. S. A. hospitals . . . . | 48 | | II | |
| Chaplains calling upon one agency to perform functions of other . . . . . . . | 26 | | II | 3 |
| Commanding Officers to bring need to attention of . . . . . . . . . . . . . . . | 26 | | II | 3 |
| Co-operation of chaplains with . . . . . | 26 | | II | 3 |
| Details regarding equipment and personnel to be settled by conference between senior representatives . . . . . . . . . | 26 | | II | 2 |
| Former to provide for amusement and recreation . . . . . . . . . . . . . . . | 26 | | II | 1 |
| Furnishing of a-sistance by one to other . | 26 | | II | 4 |
| Latter to provide for relief work . . . . . | 26 | | II | 1 |
| Partition activities non intended to give monopoly to either organization, but as a guiding rule. . . . . . . . . . . . . | 26 | | II | 2 |
| Purchasing and disbursing officers . . . . | 28 | | | |

*Le Gérant* : O. Porée. Paris — Imp. Lahure, 9, rue de Fleurus.

